# DFT calculations of ^1^H- and ^13^C-NMR chemical shifts of 3-methyl-1-phenyl-4-(phenyldiazenyl)-1H-pyrazol-5-amine in solution

**DOI:** 10.1038/s41598-022-22900-y

**Published:** 2022-10-22

**Authors:** Zaki S. Safi, Nuha Wazzan

**Affiliations:** 1 Department of Chemistry, Faculty of Science, Al Azhar University-Gaza, P.O. Box 1277, Gaza, Palestine; 2 Chemistry Department, Faculty of Science, King Abdulaziz University, P.O. Box 42805, Jeddah, 21589 Saudi Arabia

**Keywords:** Chemistry, Mathematics and computing

## Abstract

Geometries of the 3-methyl-1-phenyl-4-(phenyldiazenyl)-1H-pyrazol-5-amine azo-dye compound and its tautomer were optimized using B3LYP and M06-2X functionals in coupling with TZVP and 6–311 + G(d,p) basis sets. The ^1^H- and ^13^C-NMR chemical shifts of all species were predicted using 13 density functional theory (DFT) approaches in coupling with TZVP and 6–311 + G(d,p) basis sets at the different optimized geometries by applying the using GIAO method using the eight geometries. The selected functionals are characterized by having different amount of Hartree–Fock exchange. The selected DFT methods were B3LYP, M06-2X, BP86, B97XD, TPSSTPSS, PBE1PBE, CAM-B3LYP, wB97XD, LSDA, HSEH1PBE, PW91PW91, LC-WPBE, and B3PW91. The results obtained were compared with the available experimental data using different statistical descriptors such as root mean square error (RMSE) and maximum absolute error (MAE). Results revealed that the prediction of the ^1^H-NMR chemical shifts has more significant dependence on the applied geometry than that of the prediction of the ^13^C-NMR chemical shifts. Among all the examined functionals, B97D and TPSSTPSS functionals were found to be the most accurate ones, while the M06-2X functional is the least accurate one. Results also revealed that the prediction of NMR chemical shifts using TZVP basis sets results is more accurate results than 6–311 + G(2d,p) basis set.

## Introduction

Azo-Dyes, consisting of 60–70% of dyes, have a great importance in science and technology, such as their applicability in optical materials, molecular data processing, ion sensors, and biological applications^1^. Simultaneously, most of the azo dyes are tautomeric or potentially tautomeric^2–5^. The tautomerism phenomenon, which plays a significant role in the stability and the color of the dyes, has been extensive studied during the last decades^6–11^. The intramolecular 1,3-proton transfer process, which is very fast at the NMR time scale, occurs by the migration of a proton from the amino nitrogen atom to the carbonyl oxygen atom. Thus, to overcome this problem, an averaged signal is typically measured^7,12^. Therefore, reference compounds are needed to evaluate the proportion of the azo and hydrazo tautomers. The selection of these references that represent the pure azo and hydrazo tautomers is necessary. Thus, there is a need for such reference compounds to be identified and used in the NMR investigations of tautomeric azo dyes.

Up to now, nuclear magnetic resonance (NMR) spectroscopy has been one of the most powerful and reliable methods for structure elucidation. ^1^H- and ^13^C-NMR are the most widely studied nuclei. However, other nuclei such as ^15^ N, ^31^P, ^18^O.etc., are also under consideration, which can be attributed to the corresponding routinely available techniques readily yielding an overabundance of structural and kinetical information for the proper identification of novel compounds. Unfortunately, complex compounds suffer from the main problem in the full signal assignment, which is sometimes impossible, even with the utilization of multidimensional NMR experiments.

Calculating the nuclear magnetic shielding tensor (NMST) is required for the theoretical NMR investigations. For this reason, several computational methods were developed such as the GIAO (gauge-independent atomic orbitals)^13–17^, the IGLO (an individual gauge for localized orbital)^18^, and the CGST (continuous set of gauge transformation)^19,20^ methods. In general, the GIAO method has been shown to achieve faster convergence of the shielding value with respect to the size of the basis set and, consequently, has been the most commonly employed procedure^21^.

Last past years have witnessed a great development in the growth of quantum mechanical calculations quickly, which aids the determination of compound structures^22–24^. Different levels of theory can be effectively used to compute the NMR shielding constants. These levels include coupled-cluster theories (e.g., coupled-cluster singles–doubles (CCSD) and coupled-cluster singles–doubles–perturbative-triples, CCSD(T))^23^ and Møller-Plesset (MP) second-order perturbation theory (MP2)^22,25^. Exactly, the most accurate sets and highly comparable with the experimental data of benchmark NMR shielding constants were performed using MP2, CCSD, and CCSD(T) methods in various basis sets. Unfortunately, these methods are highly expensive and computationally time consumable, and they are only exclusively applicable to small model molecules and not for larger ones^22–25^.

In spite of being less accurate, DFT methods, on the opposing, can handle structures composed of several hundreds of atoms^26–31^. DFT methods allow the calculation of useful chemical properties, structural and electronic analysis of molecules and the theoretical values of the ^13^C- and ^1^H-NMR chemical shifts with a high degree of accuracy compared to the experimental data^32,33^. Bango et al.^27^ determined the ^13^C-NMR chemical shifts experimentally and theoretically by DFT calculations B3LYP with the 6-31G(d,p) and cc-pVTZ basis sets. Bally and Rablen^28^ proved that B3LYP/6-31G(d,p)u + 1 s (one of the most economical methods) and B3LYP/ccp-pVTZ (ca.8 times more expensive in terms of CPU time) predicted the ^1^H-NMR with root mean square of 0.5 Hz, compared to the experimental results. Broadbelt et al.^34^ accurately computed the ^1^H- and ^13^C-NMR chemical shifts using the BMK method with different basis sets in toluene-*d*_*8*_ solvent using IEFPCM solvation model. The agreement between the experimental data and the DFT computed results was improved by inclusion of vibrational corrections for DFT methods, which contrasts the behavior of the coupled-cluster methods^23,24,29,34–40^. This methodology could be applied as a promising alternative in support of traditional NMR experimental techniques. Furthermore, previous studies^40,41^ showed that DFT theory brings a respected tool for the prediction of ^13^C-NMR properties, even with a small basis set. Additionally, it was recommended to use a larger basis set, including polarization functions, for a more accurate prediction of trends in the experimental ^1^H-NMR chemical shifts^41^. Furthermore, most of studies that related to the computational of ^1^H- and ^13^C-NMR were mainly focused on DFT and ab initio performance for structures in CDCl3, which expected due to the fact that a large number of experimental spectra are taken in deuterated chloroform and there is an abundance of data for numerous molecules and functional groups.

### Test set

**Figure 1:**
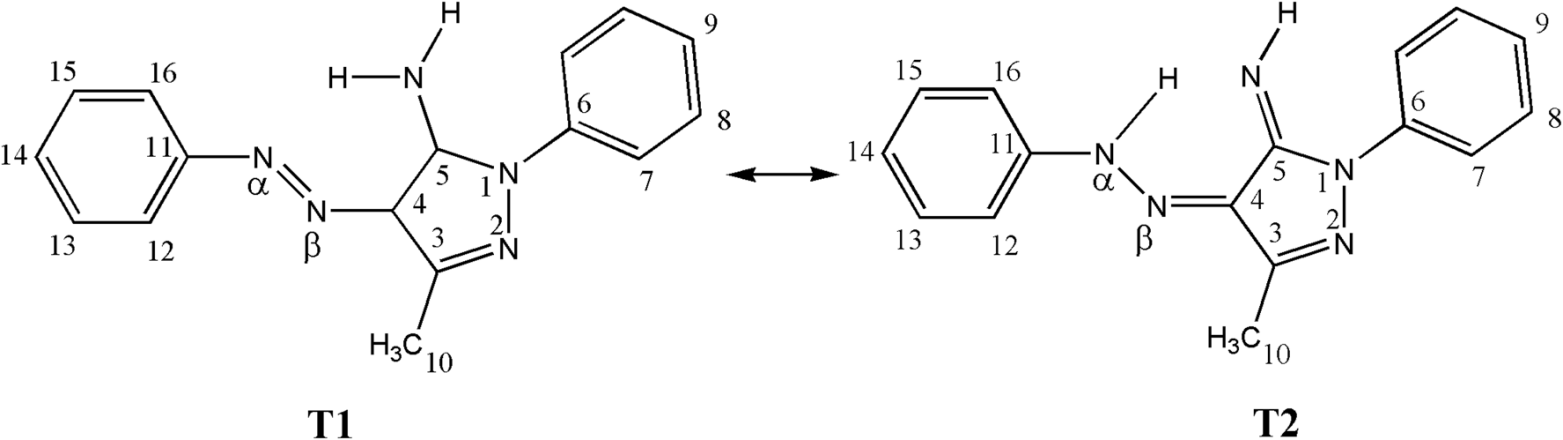
Chemical structure of 3-methyl-1-phenyl-4-(phenyldiazenyl)-1H-pyrazol-5-amine (**T1**) and its tautomer (**T2**) in ^1^H- and ^13^C-NMR test.

The 3-methyl-1-phenyl-4-(phenyldiazenyl)-1H-pyrazol-5-amine (**T1)** may have another conformer (T2) that can rapidly interconvert at the NMR time scale with relative energy of about 9.3 kcal/mol^10^ (Fig. 1). The ^1^H- and ^13^C-NMR experimental chemical shifts of the main species (**T1**) in CDCl_3_ were taken exclusively from Deneva et al.^10^. All protons that are bonded to the nitrogen atoms were not included in the validation test because they are strongly affected by the ability to form hydrogen bonds as well as the solvent environment^34^. This study is undertaken to present and discuss the following issues: (i) computation of ^1^H- and ^13^C- NMR chemical shifts for the species under probe at different geometries that optimized using B3LYP and M06-2X approaches with TZVP and 6–311 + G(2d,p) basis sets within the DFT framework using the GIAO^42^ method and (ii) statistical validation of the theoretical NMR chemical shifts based on the available experimental data.

### Computational methodology

Geometries of the investigated species were optimized using two-hybrid DFT approaches, **B**: B3LYP^43,44^ and the **M**: M06-2x (meta-GGA)^35,45^ in acetonitrile as a solvent using the Polarizable Continuum Model (the integral equation formalism variant, IEFPCM)^48^. Two basis sets were included in the optimization process, which are: (i) the triple-ζ Pople basis sets augmented with additional polarization functions 6–311 + G(2d,p)^46^ and (ii) triple zeta for valence electrons plus polarization function, TZVP^47^. Therefore, To ensure that all geometries are minima to the electronic potential energy surface, the vibrational frequencies of each species were calculated at the same levels of theories, and the absence of any imaginary frequencies was noted. The isotropic magnetic shielding tensors were computed using thirteen different XC DFT approaches, applying the gauge-independent atomic orbital (GIAO)^42^ approach in coupling with the same basis sets CHCl_3_ solvent. The classification of the thirteen XC different functionals are given in supplementary materials. In order to the simplify the following of our study, DFT approaches B3LYP and M06-2X are given the abbreviations T and P, respectively. The two bases sets TZVP and B3LYP/6–311 + G(2d,p) are given the abbreviation T and P, respectively. So that, BT, BP, MT and MP optimization levels were considered. To calculate the NMR chemical shifts, XC DFT approach is coupled with the two bases sets at the considered geometries. In total, eight combinations of theoretical approach will be used to compute the NMR chemical shifts (see Table S1 of the supporting information). All quantum chemical calculations were performed using Gaussian 09 computational software package^49^. 

Because NMR chemical shifts result from a dynamic process, the isotropic magnetic shielding tensors were averaged over all symmetry related carbons and hydrogens where applicable. For each ^1^H and ^13^C nucleus, the average isotropic magnetic tensor of the two tautomeric forms ${\sigma }_{cal}$ was taken. Then, the isotropic chemical shifts ${\delta }_{cal}$ was defined as ${\delta }_{cal}={\sigma }_{TMS}- {\sigma }_{cal}$, where ${\sigma }_{TMS}$ is the anisotropic shielding constant of ^1^H and ^13^C in trimethylsilane (TMS). Notice that ${\sigma }_{TMS}$ values of ^1^H- and ^13^C- NMR of the TMS were computed using the same strategy that used for the azo-dye species and based on the MP2 geometry (all ${\sigma }_{TMS}$ are listed in Table S2 of the supporting information). Also, it should be noted that the ${\sigma }_{TMS}$ for the ^1^H- and ^13^C-NMR were calculated as the average for the 12 hydrogen nuclei and 4 carbon atoms, respectively. The ${\delta }_{cal}$ values were collected in Tables S3 and S4 of the supporting information.

Due to the possibility of the tautomerization of the investigated species at the NMR time, the scaled ^1^H- and ^13^C-NMR chemical shifts of the hydrogen and carbon nuclei of the **T1** and **T2** species were averaged. The averaged ^1^H- and ^13^C-NMR chemical shifts were tested for possible outliers using 1.5xIRQ rule, and no outliers were detected. Computation of the different statistical descriptors was performed using Microsoft Excel 2016 using the following equations:

1 $$\left(\mathrm{Mean\,signed\,error}\right) \mathbf{M}\mathbf{E}=\frac{1}{n} \sum ({\delta }_{cal}-{\delta }_{exp})$$

2 $$\mathrm{Absolute\,difference }(\mathrm{error}) \Delta{\varvec{\delta}}= \left|{\delta }_{cal}-{\delta }_{exp}\right|$$

3 $$\mathrm{Maximum\,Absolute\,Error }\left(\mathrm{Mean\,unsigned\,error}\right): \mathbf{M}\mathbf{A}\mathbf{E}=\frac{1}{N}\sum \Delta \delta$$

4 $$\mathrm{Maximum\,difference}:\mathbf{M}\mathbf{A}\mathbf{X}=max\Delta \delta$$

5 $$\mathrm{Mean\,square\,error}:\mathbf{M}\mathbf{S}\mathbf{E}= \frac{1}{N}\sum \Delta {\delta }^{2}$$

6 $$\mathrm{Root\,mean\,square\,error}:\mathbf{R}\mathbf{M}\mathbf{S}\mathbf{E}= \sqrt{\frac{1}{N}\sum \Delta {\delta }^{2}} =\sqrt{MSE}$$

7 $$\mathrm{Mean\,absolute\,percentage\,error}:\mathbf{M}\mathbf{A}\mathbf{P}\mathbf{E}=\frac{\sum_{i=1}^{n}\left|\frac{{\delta }_{cal}- {\delta }_{exp}}{{\delta }_{exp}}\right|}{n}\times 100$$

## Results and discussion

The optimized structures of the two envisaged tautomers of the azo-dye species as obtained using B3LYP/TZVP level of theory in acetonitrile are shown in Figure S1 of the supporting information. The statistical parameters (**MAE, MAX, RMSE and MAPE**) are measured for suitable errors. Among all of the statistical descriptors, **RMSE** is the most powerful descriptor that can be used to compare the different DFT methods. The smaller the **RMSE** parameter, the more accurate the DFT approach to estimate the ^1^H- and ^13^C-NMR chemical shifts.

### Benchmark of ^1^H NMR chemical shifts

**Figure 2:**
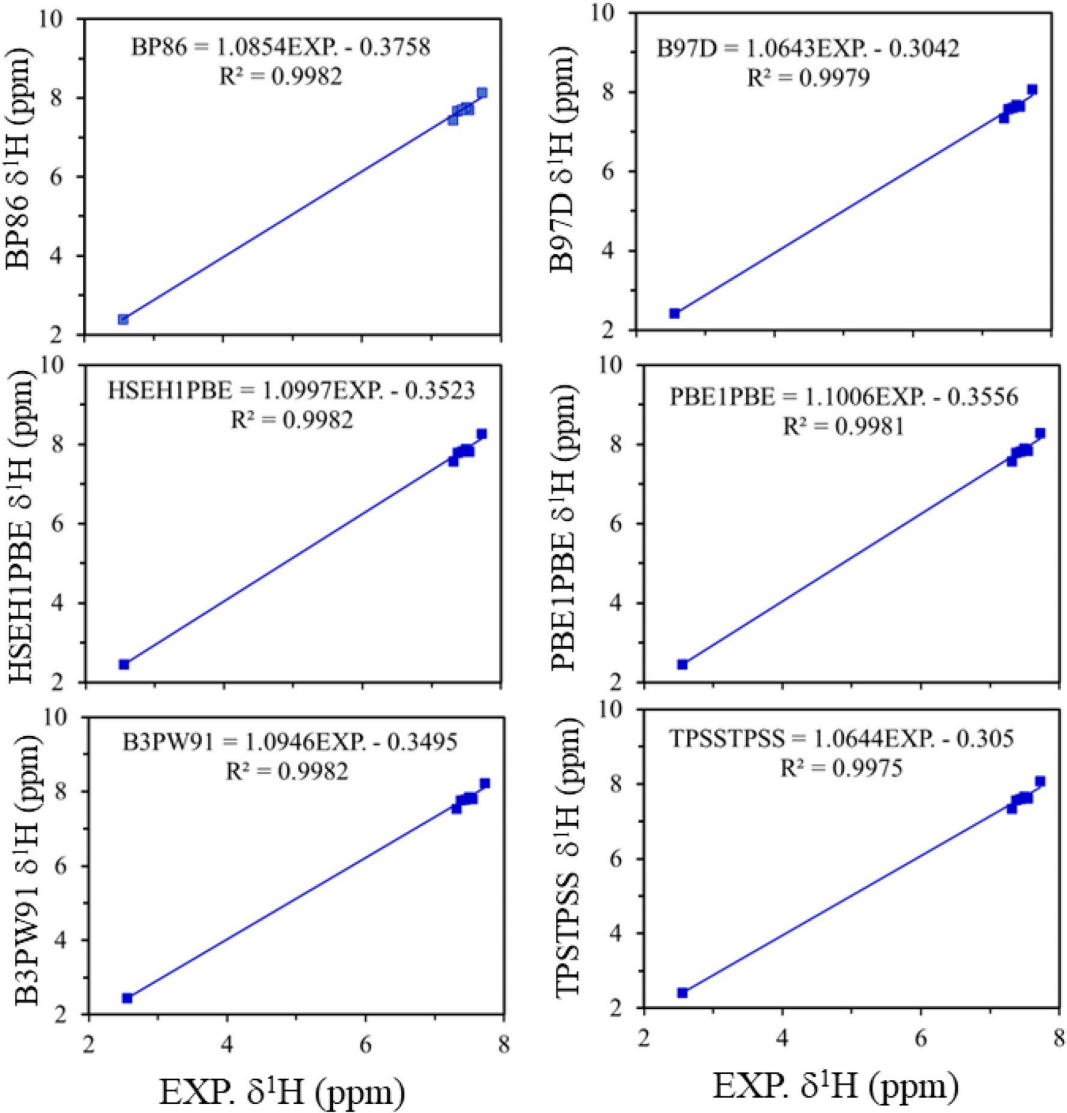
Correlation between the experimental ^1^H-NMR chemical shifts and the computed ^1^H-NMR chemical shifts as calculated using six different DFT methods (BP86, B97D, HSEH1PBE, PBE1PBE, B3PW91, and TPSSTPSS) with TZVP basis set at the B3LYP/TZVP geometry (**TBT** combination).

Tables S5 and S6 of the supporting information list all the descriptors (RMSE, MSE, MAE, MAX, MAPE, R^2^, slope, and intercept) computed for ^1^H-NMR deviations. Excellent linear regression correlation coefficient (R^2^ > 0.997–0.999) is obtained. However, the slope (0.906–1.118 ppm) and the intercept (−0.456–0.534 ppm) have slightly deviated from the ideal values for all combinations (Fig. 2 and Tables S5 and S6). The poorest regression was obtained for M06-2X functional, while the best one was obtained for TPSSTPSS and B97D functionals. Figure S1 shows graphically by column the statistical descriptors (**RMSE**, **MAX,** and **MAE**) computed for the ^1^H-NMR chemical shift deviations. For all combinations, the results show that the **MAX** descriptor (blue column), which corresponds to the maximum deviation with respect to the experimental data, is much higher than that of **RMSE** (red column) and **MAE** (dark red column) descriptors. The corresponding **MAX** arises from the computation of ^1^H-NMR chemical shifts of the hydrogen atom attached to C12 (Fig. 2). The reason may be attributed to the possible interaction (by non-covalent van der Waal) between the hydrogen atom (van der Waal radii = 2.02 Å) and the N(β) (van der Waal radii = 2.25 Å) of the azo-dye group, resulting in a weak hydrogen bond. The geometrical structure of the T1 and T2 tautomers show that the distance between the hydrogen atom and N(β) atom is an average of 2.450 Å, which strongly confirms our suggestion.

The data given in Tables S2 and S5, show that the signed deviations (ME) are typically positive, indicating that the computed ^1^H NMR chemical shifts are exclusively higher than that of the experimental ones. It is also found that ~ 93.5% of the ME values are positive. The number of negative ME values are ~ 96 out of 1456, with a percentage of ~ 6.5%. Moreover, most of the negative values belong to the hydrogen atom attached to C10 atom, which may be attributed to the consideration of the average value of the isotropic magnetic tensors of the three hydrogen atoms attached to C10.

### Basis set effect

In order to analyze the effect of the basis set that couples with the XC functional on the 1H-NMR chemical shift, two basis sets TZVP and 6–311 + G(2d,p) were selected. For the sake of comparison, the eight combinations (Table S4) were divided into two parts based on the basis sets, which coupled with NMR XC functional, as follows:

- Part (1) in which the NMR functional combined with TZVP basis set, and it includes C1, C3, C5 and C7 combinations, and
- Part (2) in which the NMR functional combined with 6–311 + G(d,p) basis set, includes C2, C4, C6, and C8 combinations.

**Figure 3:**
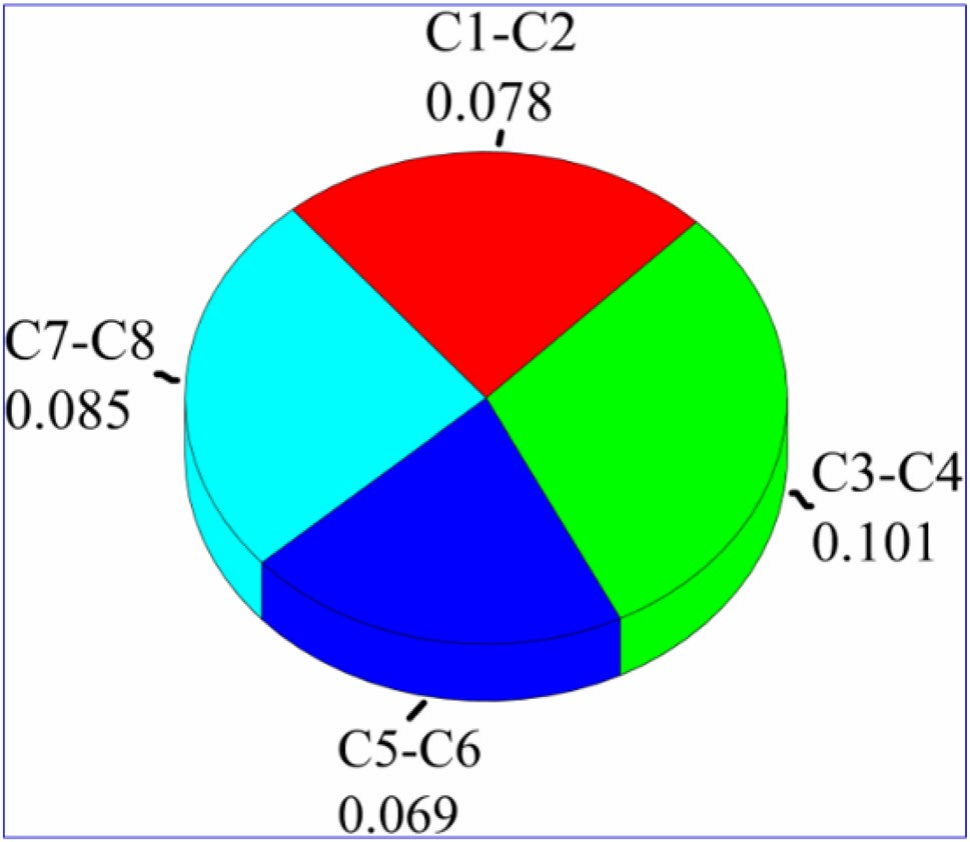
3-D color Pie chart of the average difference of RMSE for ^1^H-NMR chemical shifts.

To simplify the discussion, the average absolute differences in **RMSE** values were considered and plotted as pie charts (Fig. 3). In this case, changing the basis set coupled with the XC functionals to compute the 1H-NMR chemical shifts is considered. Taking into account the same geometry level, the results show that the accuracy of the ^1^H-NMR chemical shifts as computed by TZVP basis set is higher than that computed by 6–311 + (2d,p) basis set. Actually, it is found that the results obtained by TZVP basis set are 0.069–0.101 ppm higher than those obtained by 6–311 + G(2d,p) one. Moreover, the highest differences are found in the case of using 6–311 + G(2d,p) basis set in the optimization process (combinations C3, C4, C7 and C8). These results led us to conclude that the accuracy of the computed ^1^H-NMR values using TVZP basis set is much higher than that of 6–311 + G(2d,p) one na dit can be considered as the best choice to compute the ^1^H-NMR chemical shifts.

### Geometry level effect

**Figure 4:**
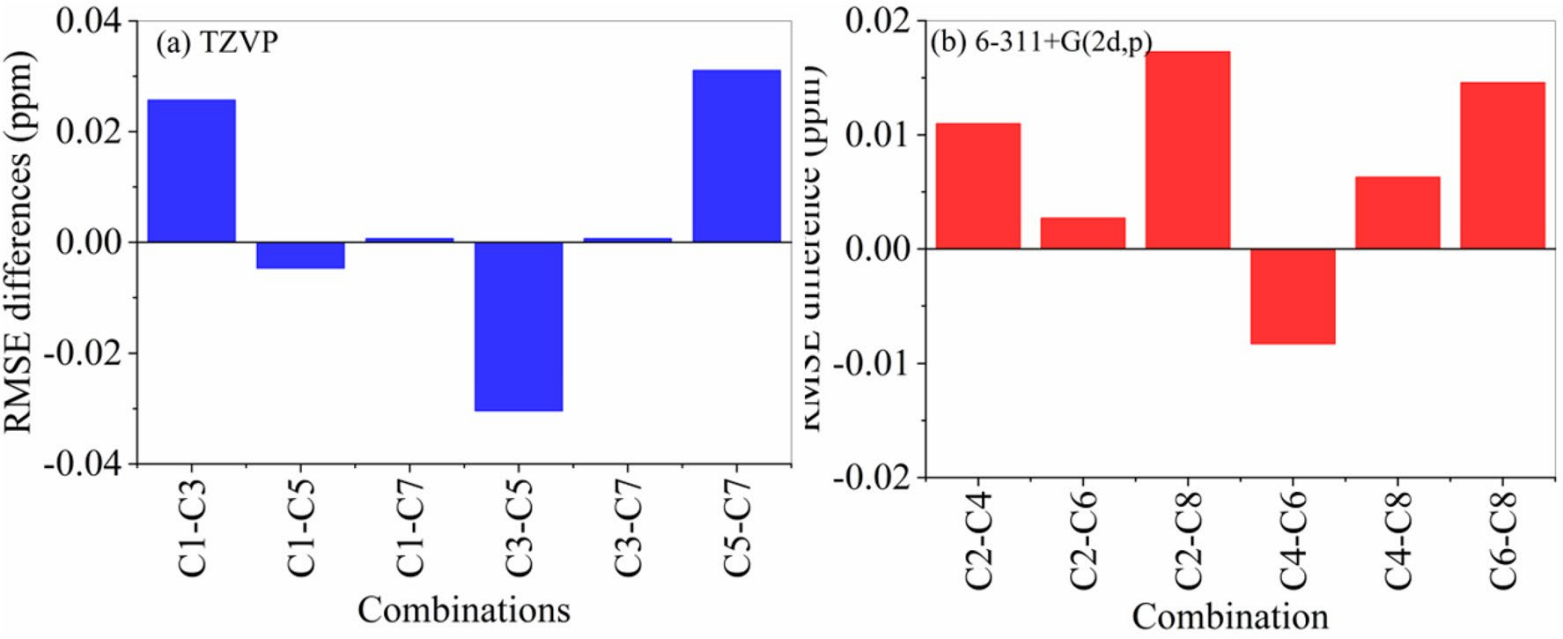
Graphical representation of the average difference of RMSE values (in ppm) between each pair of the eight combinations (**a**) TZVP and (**b**) 6–311 + G(2d,p). C1-C8 belong to the combination numbers from 1–7.

Four different geometrical structures were optimized using **BT**, **MT**, **BP,** and **MP** levels of theories (Table S1). Figure 4 shows the graphical representation by columns for the average differences of the **RMSE** values obtained by taking into account the same basis sets that are coupled with the different XC functionals. Computation of the ^1^H-NMR chemical shifts using DFT/TZVP at B3LYP/6–311 + G(2d,p) geometries (Combination 3) is more accurate than those at B3LYP/TZVP geometries (C1) (Fig. 4a). Whereas the computed ^1^H-NMR chemical shifts at M06-2X/6–311 + (2d,p) geometry (combination 5) are more accurate than those at the M06-2X/TZVP geometry (Combination 7). On the other hand, using B3LYP/6–311 + G(2d,p) geometry can compute more accurate ^1^H-NMR chemical shifts than the M06-2X/TZVP geometries (combinations **C3** and **C5).** Similar results can also be found for the case of DFT/6–311 + G(2d,p) NMR level at the four geometries (Fig. 4b).

Based on the above results, one may conclude that taking into account either M06-2X or B3LYP functionals, using of the 6–311 + G(2d,p) basis set in the optimization steps computes accurate more accurate ^1^H-NMR chemical shifts than the TZVP basis sets.

### XC functionals effect

Besides to the basis set and geometry level effects, one of the main questions of this study is whether the DFT functionals can compute the most accurate ^1^H-NMR chemical shifts? The answer of this question can be achieved by a careful looking at the results obtained indicates, in general, that M06-2X, LC-WPBE, and wB97XD functionals compute the poorest ^1^H-NMR chemical shifts with very high RMSE values (see Tables S5 and S6 and shown in Figure S1). Whereas, the highest degree of accuracy is obtained by B97D, TPSSTPSS, and BP86 functionals, which is in an excellent agreement with the literature^50^. This finding can be attributed to the amount of HF exchange in the functionals used: (M06-2X (54%), LSDA (45%), and wB97XD (25%)^50^.

### Combination effect

**Figure 5:**
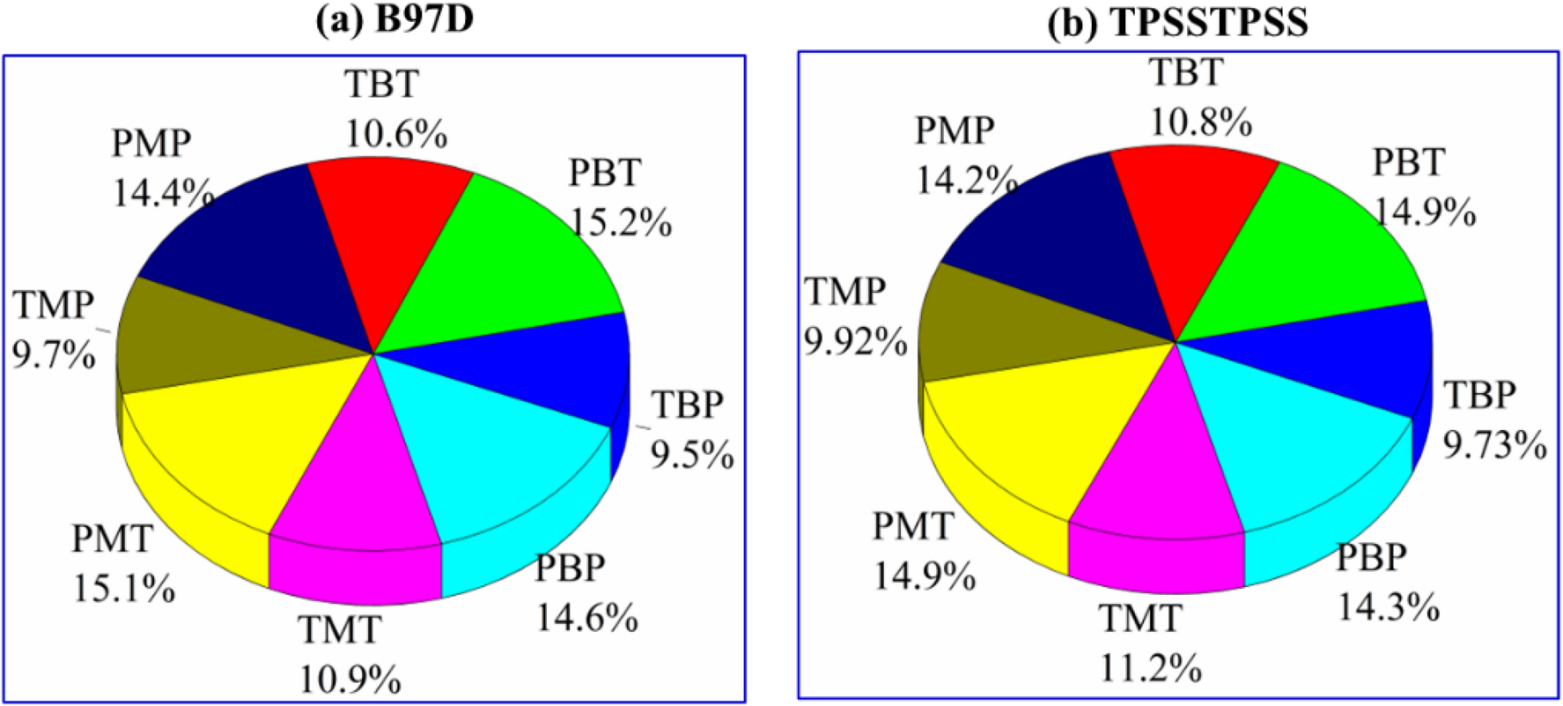
3-D color Pie chart of the computed root mean square error (RMSE) for the ^1^H-NMR chemical shifts of the 3-methyl-1-phenyl-4-(phenyldiazenyl)-1H-pyrazol-5-amine compound as obtained using B97D (**a**) and TPSSTPSS (**b**) functionals based on eight different combinations of geometry and bases sets.

Eight combinations (C1-C8) have been considered (Table S1**)** to compute the ^1^H-NMR chemical shifts. To shed some light on the effect of the several combinations on the accuracy of the computed ^1^H-NMR chemical, Fig. 5a,b shows the RMSE% of the B97D and TPSSTPSS functionals. It is clearly seen that the most accurate results are obtained for the DFT approach with the **TBP** combination (**C3**). Indeed, the **RMSE%** are 9.5% and 10.8% for B97D and TPSSTPSS functionals, respectively. Whereas the least accurate results were computed using the DFT apprroach with **PBT** combination (C4), with **RMSE%** of 15.2% and 14.9% for B97D and TPSSTPSS functionals, respectively. These results led us to safely conclude that the ^1^H-NMR chemical shifts, with a high degree of accuracy, can be computed by either B97D or TPSSTPSS functionals in coupling with **TBP.**

To get an exclusive order for the different DFT approaches, the computed descriptors obtained for the **TBT, TBP, TMT** and **TMP** combinations were averaged, and the results are listed in Table 1. The most important statistical descriptors (RMSE, MAX, MAE, and MAPE) are graphically shown in Figure S3. It is clearly obvious from Table 1 and Figure S3 that the corresponding RMSE, MAX, MAE, MSE, ME and MAPE descriptors are 0.006 (1.6%), 0.020 (3.5%), 0.006 (1.6%), 0.004 (2.8%), 0.008 (2.7%) and 0.002 (3.4%) ppm, respectively. These results indicate that there are no significant differences in the computed ^1^H-NMR chemical shifts based on using either B3LYP or M06-2X geometry. These findings led us to conclude that the main factors that can influence the calculation of the ^1^H-NMR chemicals are the DFT approach and the selected basis sets, regardless of the geometry level used.

**Table 1:** The averaged descriptors RMSE, MAE, ME, MAX, MAPE, ME, R^2^, slope, and intercept computed for ^1^H-NMR deviations for each combination of geometry level, basis set, and functional. The table is sorted by RMSE column.

Functional	RMSE	MAX	MAE	MSE	ME	MAPE	R^2^	slope	intercept
**B3LYP**									
B97D	0.182	0.333	0.157	0.033	0.116	2.58%	0.9978	1.064	0.312
TPSSTPSS	0.184	0.341	0.157	0.034	0.115	2.59%	0.9976	1.064	0.316
BP86	0.252	0.407	0.234	0.064	0.187	3.48%	0.9981	1.085	0.370
PW91PW91	0.272	0.417	0.255	0.074	0.207	3.78%	0.9983	1.091	0.386
B3LYP	0.289	0.484	0.263	0.084	0.238	3.52%	0.9977	1.079	0.291
B3PW91	0.310	0.476	0.290	0.097	0.252	3.98%	0.9982	1.093	0.357
HSEH1PBE	0.361	0.539	0.336	0.130	0.307	4.37%	0.9982	1.099	0.340
PBE1PBE	0.365	0.548	0.339	0.133	0.311	4.42%	0.9981	1.099	0.341
LSDA	0.380	0.539	0.361	0.144	0.314	4.87%	0.9983	1.115	0.427
CAM-B3LYP	0.384	0.599	0.353	0.148	0.332	4.50%	0.9976	1.099	0.321
WB97XD	0.428	0.575	0.401	0.183	0.375	4.99%	0.9985	1.111	0.347
LC-WPBE	0.527	0.691	0.492	0.278	0.471	5.99%	0.9987	1.130	0.373
M6-2X	0.837	1.140	0.772	0.701	0.772	9.16%	0.9971	1.175	0.372
**M06-2X**									
B97D	0.187	0.352	0.161	0.035	0.111	2.73%	0.9976	1.069	0.348
TPSSTPSS	0.189	0.359	0.161	0.036	0.109	2.73%	0.9973	1.069	0.352
BP86	0.254	0.425	0.235	0.065	0.182	3.63%	0.9979	1.090	0.404
PW91PW91	0.261	0.434	0.242	0.068	0.187	3.72%	0.9979	1.092	0.413
B3LYP	0.289	0.499	0.262	0.084	0.230	3.65%	0.9974	1.084	0.329
B3PW91	0.329	0.516	0.307	0.108	0.269	4.22%	0.9980	1.098	0.370
PBE1PBE	0.363	0.563	0.339	0.132	0.303	4.56%	0.9979	1.104	0.378
HSEH1PBE	0.373	0.571	0.347	0.140	0.316	4.57%	0.9980	1.103	0.361
LSDA	0.380	0.556	0.363	0.145	0.309	5.02%	0.9983	1.120	0.459
CAM-B3LYP	0.394	0.618	0.362	0.156	0.339	4.67%	0.9975	1.103	0.341
WB97XD	0.423	0.588	0.399	0.179	0.366	5.11%	0.9987	1.116	0.382
LC-WPBE	0.523	0.704	0.491	0.274	0.462	6.14%	0.9987	1.136	0.411
M6-2X	0.831	1.122	0.765	0.691	0.765	8.93%	0.9977	1.179	0.396

Taken into account the B3LYP geometry, the accuracy of the different functionals can be ranked as follows: TPSSTPSS > B97D > BP86 > PW91PW91 > B3LYP > B3PW91 > HSEH1PBE > PBE1PBE > LSDA > CAM-B3LYP > wB97XD > LC-WAPBE > M06-2X. Whereas, taken into account the M06-2X geometry, the accuracy of the different functionals can be ranked as follows: B97D > TPSSTPSS > BP86 > PW91PW91 > B3LYP > B3PW91 > PBE1PBE > HSEH1PBE > LSDA > CAM-B3LYP > wB97XD > LC-WAPBE > M06-2X.

### Benchmark of ^13^C-NMR chemical shifts

**Figure 6:**
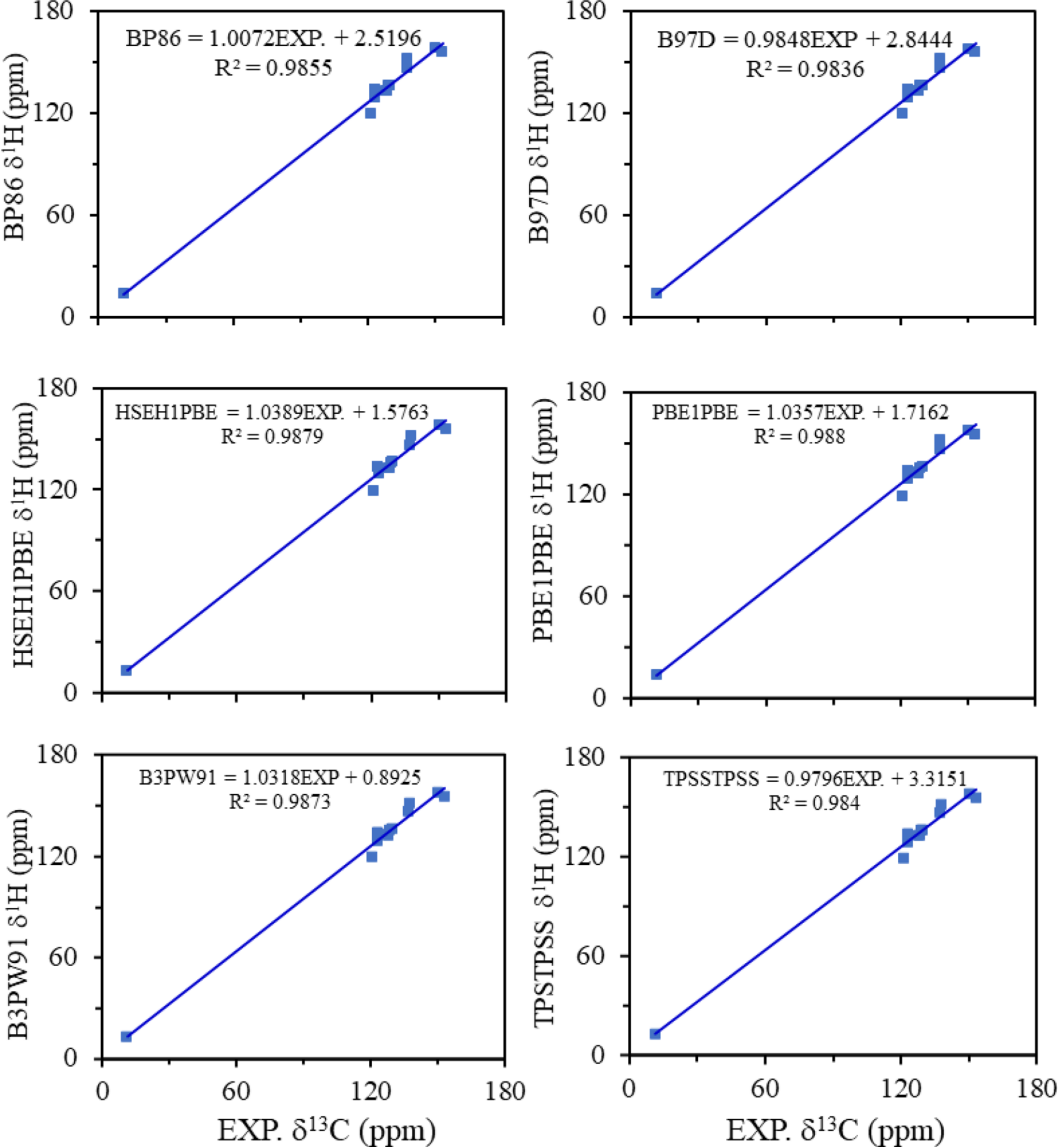
Correlation between the experimental ^13^C-NMR chemical shifts and the computed ^13^C-NMR chemical shifts as calculated using six different DFT methods (BP86, B97D, HSEH1PBE, PBE1PBE, B3PW91, and TPSSTPSS) with TZVP basis set at the B3LYP/TZVP geometry (**TBT** combination).

In the following, we are going through the analysis of the results obtained for the prediction of ^13^C-NMR. Tables S7 and S8 list all the statistical descriptors (RMSE, MSE, MAE, MAX, MAPE, R^2^, slope and intercept) computed for ^13^C-NMR deviations. An excellent linear regression correlation coefficient (R^2^ > 0.986–0.993) was obtained. It is also found that, however, the slope (0.963–1.149 ppm), 84% of these values are in agreement with the ideal values of slope (Fig. 6). The intercept (0.342–3.786 ppm) differed slightly from the ideal values for all combinations (see Tables S5 and S6). The best regressions are obtained for TPSSTPSS and B97D functionals, while the worst ones are obtained for the M06-2X functional.

**Figure 7:**
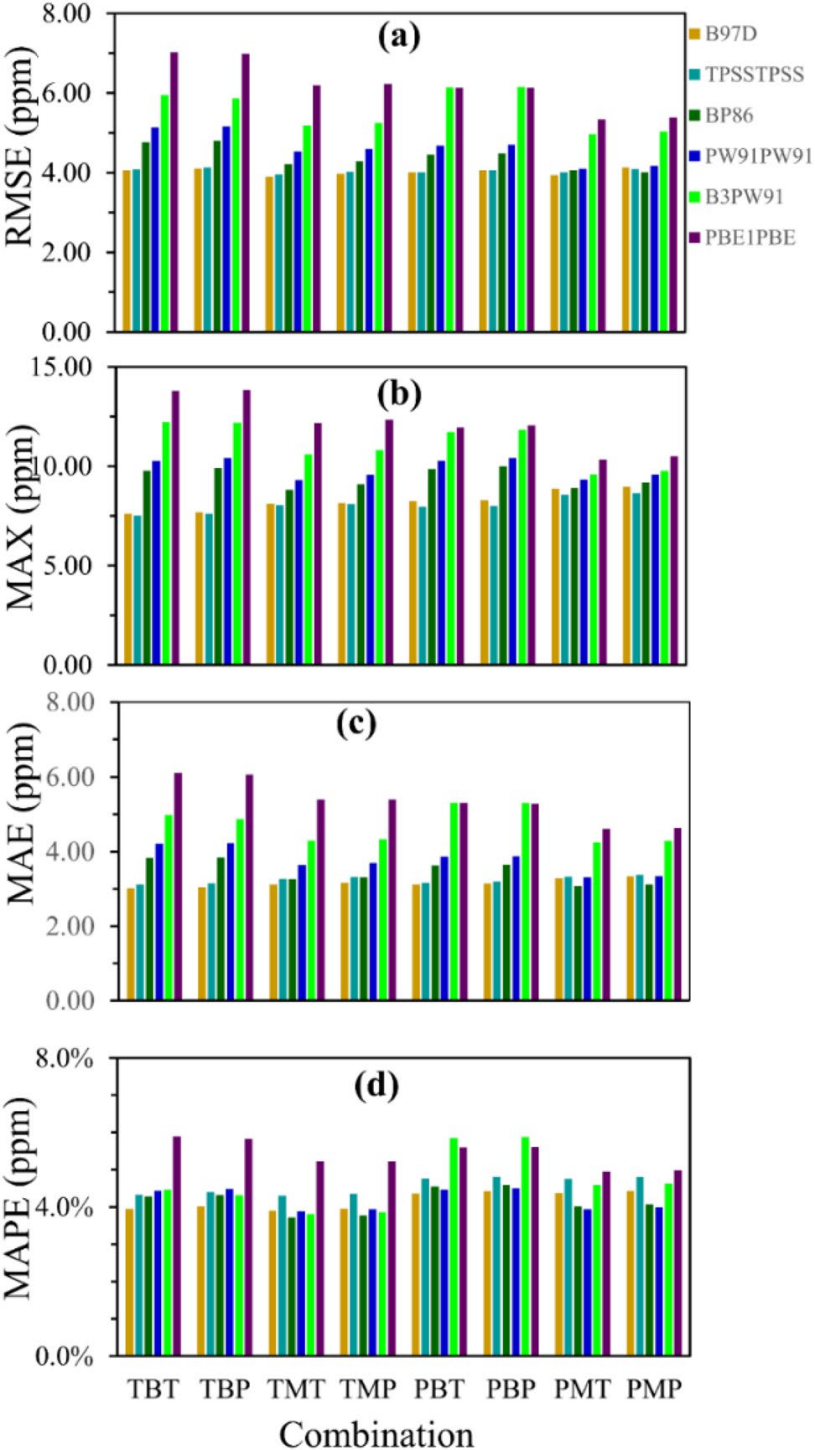
Graphical representation of statistical descriptors ((**a**) RMSE, (**b**) MAX, (**c**) MAE and (**d**) MAPE) computed for ^13^C deviations versus each combination of geometry and basis set. Notice: T and P denote TZVP basis sets, B and M correspond to B3LYP and M06-2X DFT functionals used for the geometry optimization process.

Figure 7 shows only the statistical descriptors (RMSE, MAX, MAE, and MAPE) for the best ^13^C-NMR results (B97D, TPSSTPSS, BP86, B3PW91, PBE1PBE and PW91PW91 functionals), which agrees with the previous results^39^. With the exception to the results of wB97XD and CAM-B3LYP functionals, the accuracies of the different DFT approaches that were used to compute the ^1^H- and ^13^C-NMR chemical shifts are almost the same. Therefore, the most accurate ^13^C-NMR chemical shifts are computed using B97D, TPSSTPSS, and BP86 functionals. Whereas the least accurate results are obtained using M06-2X, LC-WPBE, and CAM-B3LYP functionals.

### Combination effect

**Figure 8:**
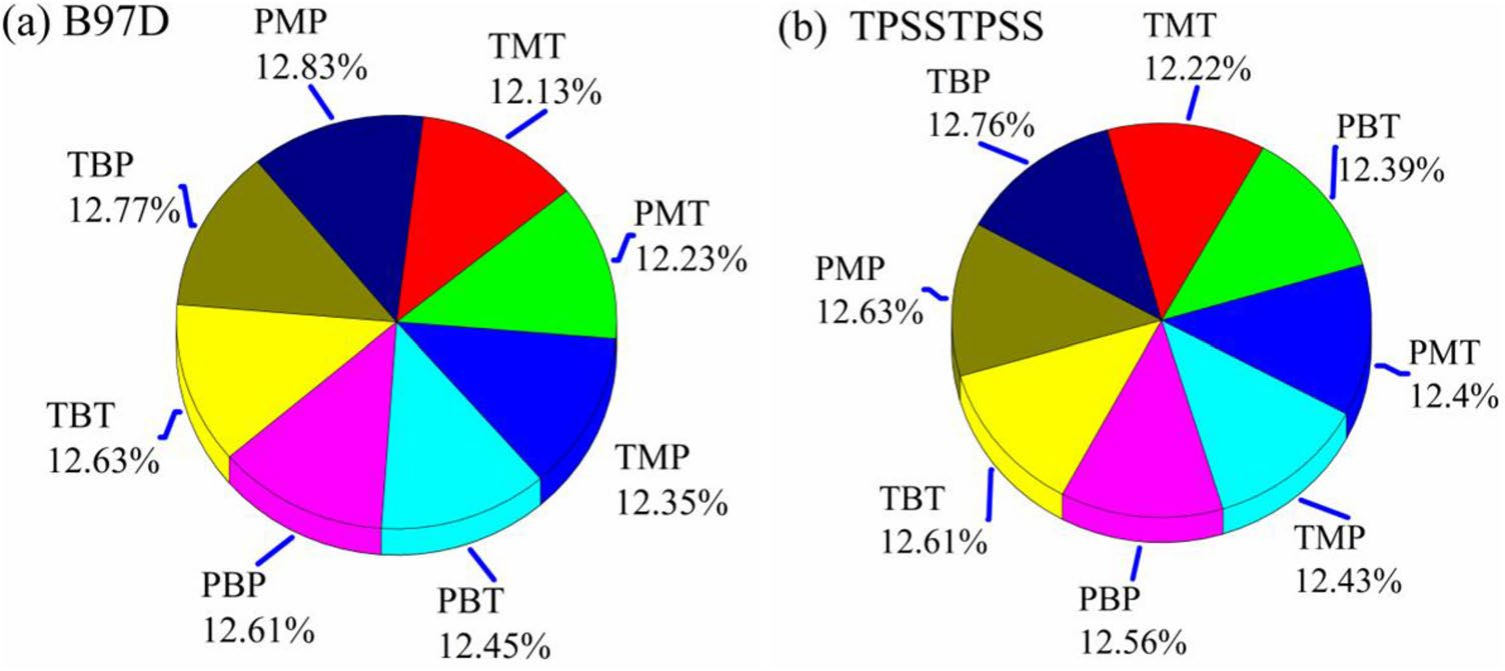
A 3D color pie chart for the computed RMSEs computed by (**a**) B97D and (**b**) TPSSTPSS functionals versus the different combinations (geometry level and basis set) (Notice: T and P correspond, respectively, to TZVP and 6–311 + G(2d,p), B and M correspond to B3LYP and M06-2X geometry functionals).

To analyze the effect of the different combinations (C1-C8) on the accuracy of the ^13^C-NMR chemical shifts, we apply the same scenario we used for ^1^H-NMR results. In comparison with the experimental results^10^, Fig. 8 shows the 3D color pie charts of the RMSE computed for the ^13^C-NMR chemical shifts obtained using B97D and TPSSTPSS, BP86, and PW91PW91. For both functionals, the least deviations of 12.13 and 12.22% (TPSSTPSS)) were computed using the B97D/TZVP//M06-2X/TZVP and TPSSTPSS/TZVP//M06-2X/TZVP levels, respectively, while the highest deviations (least accurate) were computed at 12.83 and 12.76% were computed using the B97D/6–311 + G(2d,p)//M06-2X/6–311 + G(2d,p) and TPSSTPSS/TZVP//B3lYP/6–311 + G(2d,p) levels, respectively. Based on these results, one concludes that the prediction of the ^13^C NMR chemical shifts can be accurately computed by using the D97D and/or TPSSTPSS in coupling with the TZVP basis set at the M06-2X/TZVP geometry.

To rank the different DFT approaches that we have used to compute the ^13^C-NMR chemical shifts, the data in Tables S7 and S8 were averaged and classified into two main combinations. The first one averages the computed descriptors based on the B3LYP geometry with the TZVP and 6–311 + G(2d,p) basis sets, while the second one averages the computed descriptors based on M06-2X geometry with the TZVP and 6–311 + G(2d,p) basis sets. The results are listed in Table 2, and the data are sorted by RMSE descriptors.

**Table 2:** Averaged statistical descriptors RMSE, MAX, MAE, MSE, ME MAPE, and determination coefficient (R^2^) computed for ^13^C NMR deviations obtained for each geometry and functional combination. The table is sorted by RMSE column.

Geometry	NMR Functional	RMSE	MAX	MAE	MSE	ME	MAPE	R^2^
B3LYP	TPSSTPSS	4.07	7.77	3.16	16.59	0.01	4.58%	0.9867
	B97D	4.17	8.39	3.21	17.39	0.74	4.23%	0.9873
	BP86	4.52	9.46	3.61	20.51	1.95	4.39%	0.9882
	PW91PW91	4.92	10.35	4.04	24.28	3.22	4.47%	0.9891
	B3PW91	6.02	12.05	5.11	36.27	4.58	5.06%	0.9890
	PBE1PBE	6.57	12.86	5.70	43.34	5.28	5.80%	0.9892
	HSEH1PBE	6.80	13.17	5.91	46.37	5.54	5.86%	0.9892
	B3LYP	7.19	14.00	6.27	51.79	5.89	6.32%	0.9883
	WB97XD	7.79	15.61	6.77	61.02	6.52	6.61%	0.9881
	LSDA	8.37	14.66	7.38	70.03	7.33	6.22%	0.9904
	CAM-B3LYP	9.56	18.17	8.39	91.52	8.39	7.59%	0.9883
	LC-WPBE	10.99	19.96	9.92	121.15	9.92	9.05%	0.9884
	M6-2X	20.17	25.36	19.19	407.24	19.19	16.40%	0.9915
M06-2X	B97D	4.02	8.53	3.23	16.14	-0.75	4.17%	0.9877
	TPSSTPSS	4.02	8.33	3.32	16.17	-0.72	4.56%	0.9877
	BP86	4.11	9.00	3.19	16.94	1.97	3.90%	0.9894
	PW91PW91	4.35	9.45	3.49	18.97	2.47	3.94%	0.9898
	B3PW91	5.11	10.19	4.29	26.09	3.56	4.22%	0.9900
	PBE1PBE	5.79	11.34	5.01	33.66	4.48	5.10%	0.9902
	B3LYP	6.36	12.36	5.54	40.51	5.06	5.66%	0.9894
	HSEH1PBE	6.55	12.22	5.74	44.41	5.37	6.03%	0.9902
	WB97XD	6.93	13.94	6.04	48.39	5.69	5.95%	0.9894
	LSDA	7.64	13.77	6.73	58.30	6.59	5.63%	0.9909
	CAM-B3LYP	8.77	16.62	7.69	76.95	7.66	6.97%	0.9896
	LC-WPBE	10.08	18.23	9.08	101.97	9.08	8.28%	0.9898
	M6-2X	19.13	23.46	18.21	366.55	18.21	15.46%	0.9923

For the B97D, TPSSTPSS, BP86, PW91PW91and B3PW91, the average statistical errors will be as follows: RMSE = 4.74 and 4.32 ppm, MAX = 9.60 and 9.10 ppm, MAE = 3.83 and 3.50 ppm, ME = 2.10 and 1.31 ppm, and MAPE = 4.55 and 4.16% for B3LYP and M06-2X geometries, respectively. These results strongly confirm that the prediction of the ^13^C-NMR chemical shifts on the base of the M06-2X geometry, regardless of the NMR functional and basis set, is more accurate than the B3LYP geometry.

Finally, the data given in Tables S7 and S8, show that the signed deviations (ME) are typically positive, indicating that the computed ^13^C NMR chemical shifts are exclusively higher than the experimental ones. It is found that > 87% of the ME values are positive. Indeed, the number of negative ME equals approximately 340 out of 2496 values, with a percentage of ~ 13%). The maximum number of the negative ME values was computed for TPSSTPSS functionals with a percentage of ~ 40%.

## Conclusions

Eight different combinations of DFT functionals, geometry level, and basis sets have been examined for the prediction of 1Hand 13C-NMR chemical shifts in 3-methyl-1-phenyl-4-(phenyldiazenyl)-1H-pyrazol-5-amine. The computed results have been predicted using experimental shifts as a reference using several statistical descriptors. From the calculated data, the following remarked conclusion can be extracted:

- Excellent linear correlations have been obtained between the DFT-calculated and experimental ^1^H- and 13C NMR chemical shifts for all functional, basis set, and geometry level tested.
- The slopes of the linear correlations are within the ideal value for the ^13^C-NMR chemical, and it is slightly deviated in the case of ^1^H-NMR chemical shifts.
- The accuracy of the ^1^H-NMR chemical shifts computed using the TVZP basis set on the four suggested geometries was higher than those obtained by the 6–311 + G(2d,p) basis set.
- The B97D, TPSSTPSS, and BP86 functionals can compute the ^1^H- and 13C-NMR chemical shifts with a reasonable degree of accuracy.
- For ^13^C-NMR chemical shifts, the least errors have been obtained using the combination of B97D and TPSSTPSS functionals with TZVP basis sets with taken into account M06-2X/TZVP geometry.
- B97D, TPSSTPSS, and BP86 functionals are excellent choices to calculate the NMR chemical shifts with highly reasonable accuracy.
- M06-2X, LC-WPBE, WB97XD, and CAM-B3LYP are the poor functional choice to predict either 1H- or 13C- NMR chemical shifts.
- Both TZVP and 6–311 + G(2d,p) basis sets are very good choices to predict the NMR chemical shift. However, the TZVP basis set can predict the NMR shifts with a higher degree of accuracy.

## Supplementary Information

Supplementary Information 1.

Supplementary Information 2.

## Data Availability

All data generated or analyzed during this study are included in this published article [supporting information-updated]. Additionally, the datasets used and/or analyzed during the current study are available from the corresponding author upon reasonable request.

## References

[1] Zollinger H (2003). Color chemistry: Syntheses, properties, and applications of organic dyes and pigments.

[2] Kelemen J (1981). Azo-hydrazone tautomerism in azo dyes. I. A comparative study of 1-phenylazo-2-naphthol and 1-phenylazo-2-naphthylamine derivatives by electronic spectroscopy. Dyes Pigm..

[3] Kelemen J, Moss S, Sauter H, Winkler T (1982). Azo—hydrazone tautomerism in azo dyes. II. Raman, NMR and mass spectrometric investigations of 1-phenylazo-2-naphthylamine and 1-phenylazo-2-naphthol derivatives. Dyes Pigm..

[4] Kelemen J, Kormany G, Rihs G (1982). Azo—hydrazone tautomerism in azo dyes. III. The tautomeric structure of 1-(4′-nitrophenylazo)-2-naphthylamine from crystal structure determination. Dyes Pigm..

[5] Kelemen J, Moss S, Glitsch S (1984). Azo-hydrazone tautomerism in azo dyes. IV. Colour and tautomeric structure of adsorbed 1-phenylazo-2-naphthylamine and 1-phenylazo-2-naphthol dyes. Dyes Pigm..

[6] Safi ZS, Alhendawi HM (2009). Tautomerization and substituent effects on the intramolecular hydrogen bonding in 4-formyl-1-methylpyrazol-5-ol A density functional theory. Asian J. Chem..

[7] Kleinpeter, E. NMR spectroscopic study of tautomerism in solution and in the solid state. *Tautomerism: methods and theories*, 103–143 (2013).

[8] Faris WM, Safi ZS (2014). Theoretical investigation of tautomerism stability of hydantoin in the gas phase and in the solution. Orient. J. Chem..

[9] Safi ZS (2018). Conceptual density functional theory and its application in the chemical domain.

[10] Deneva V (2019). Tautomerism in azo dyes: Border cases of azo and hydrazo tautomers as possible NMR reference compounds. Dyes Pigm..

[11] Wazzan N, Safi Z, Al-Qurashi O (2020). DFT investigation on the linear and nonlinear optical properties of the tautomers and derivatives of 2-aminobenzothiazole (ABT) in the gas phase and different solvents. J. King Saud Univ.-Sci..

[12] Nedeltcheva D, Antonov L, Lycka A, Damyanova B, Popov S (2009). Chemometric models for quantitative analysis of tautomeric Schiff bases and azo dyes. Curr. Org. Chem..

[13] Wiitala KW, Hoye TR, Cramer CJ (2006). Hybrid density functional methods empirically optimized for the computation of 13C and 1H chemical shifts in chloroform solution. J. Chem. Theory Comput..

[14] Wolinski K, Hinton JF, Pulay P (1990). Efficient implementation of the gauge-independent atomic orbital method for NMR chemical shift calculations. J. Am. Chem. Soc..

[15] Pankratyev EY, Tulyabaev AR, Khalilov LM (2011). How reliable are GIAO calculations of 1H and 13C NMR chemical shifts? A statistical analysis and empirical corrections at DFT (PBE/3z) level. J. Comput. Chem..

[16] Pulay P, Hinton J, Wolinski K (1993). Nuclear magnetic shieldings and molecular structure.

[17] Grimblat N, Sarotti AM (2016). Computational chemistry to the rescue: Modern toolboxes for the assignment of complex molecules by GIAO NMR calculations. Chem.–A Eur. J..

[18] Schindler M, Kutzelnigg W (1983). Theory of magnetic susceptibilities and NMR chemical shifts in terms of localized quantities: IV. Some small molecules with multiple bonds (N_2_, HCN, CO, C_2_H_2_, CO_2_, N_2_O, O_3_, FNO). Mol. Phys..

[19] Keith T, Bader R (1992). Calculation of magnetic response properties using atoms in molecules. Chem. Phys. Lett..

[20] Keith TA, Bader RF (1993). Calculation of magnetic response properties using a continuous set of gauge transformations. Chem. Phys. Lett..

[21] Claridge TD (2016). High-resolution NMR techniques in organic chemistry.

[22] Loibl S, Schütz M (2012). NMR shielding tensors for density fitted local second-order Møller-Plesset perturbation theory using gauge including atomic orbitals. J. Chem. Phys..

[23] Teale AM, Lutnæs OB, Helgaker T, Tozer DJ, Gauss J (2013). Benchmarking density-functional theory calculations of NMR shielding constants and spin–rotation constants using accurate coupled-cluster calculations. J. Chem. Phys..

[24] Flaig D (2014). Benchmarking hydrogen and carbon NMR chemical shifts at HF, DFT, and MP2 levels. J. Chem. Theory Comput..

[25] Maurer M, Ochsenfeld C (2013). A linear-and sublinear-scaling method for calculating NMR shieldings in atomic orbital-based second-order Møller-Plesset perturbation theory. J. Chem. Phys..

[26] Ochsenfeld C, Kussmann J, Koziol F (2004). Ab initio NMR spectra for molecular systems with a thousand and more atoms: A linear-scaling method. Angew. Chem..

[27] Bagno A, Rastrelli F, Saielli G (2003). Predicting 13C NMR spectra by DFT calculations. J. Phys. Chem. A.

[28] Bally T, Rablen PR (2011). Quantum-chemical simulation of 1H NMR spectra. 2. Comparison of DFT-based procedures for computing proton–proton coupling constants in organic molecules. J. Org. Chem..

[29] Li S, Zhou W, Gao H, Zhou Z (2012). Density functional theory study of 13C NMR chemical shift of chlorinated compounds. Magn. Reson. Chem..

[30] Venianakis T (2020). DFT Calculations of 1H-and 13C-NMR chemical shifts of geometric isomers of conjugated linoleic acid (18: 2 ω-7) and model compounds in solution. Molecules.

[31] Pierens GK, Venkatachalam T, Reutens DC (2016). Comparison of experimental and DFT-calculated NMR chemical shifts of 2-amino and 2-hydroxyl substituted phenyl benzimidazoles, benzoxazoles and benzothiazoles in four solvents using the IEF-PCM solvation model. Magn. Reson. Chem..

[32] Lee C, Yang W, Parr RG (1988). Development of the Colle-Salvetti correlation-energy formula into a functional of the electron density. Phys. Rev. B.

[33] Barreiro EJ, Rodrigues CR, Albuquerque MG, Sant'Anna CMRD, Alencastro RBD (1997). Modelagem molecular: Uma ferramenta para o planejamento racional de fármacos em química medicinal. Química nova.

[34] Konstantinov IA, Broadbelt LJ (2011). Regression formulas for density functional theory calculated 1H and 13C NMR chemical shifts in toluene-d 8. J. Phys. Chem. A.

[35] Zhao Y, Truhlar DG (2008). Improved description of nuclear magnetic resonance chemical shielding constants using the M06-L meta-generalized-gradient-approximation density functional. J. Phys. Chem. A.

[36] Stoyanov SR, Gusarov S, Kuznicki SM, Kovalenko A (2008). Theoretical modeling of zeolite nanoparticle surface acidity for heavy oil upgrading. J. Phys. Chem. C.

[37] Grimme S (2017). Fully automated quantum-chemistry-based computation of spin–spin-coupled nuclear magnetic resonance spectra. Angew. Chem. Int. Ed..

[38] Stoychev GL, Auer AA, Neese F (2018). Efficient and accurate prediction of nuclear magnetic resonance shielding tensors with double-hybrid density functional theory. J. Chem. Theory Comput..

[39] Iron MA (2017). Evaluation of the factors impacting the accuracy of 13C NMR chemical shift predictions using density functional theory the advantage of long-range corrected functionals. J. Chem. Theory Comput..

[40] Pierens GK, Venkatachalam T, Reutens DC (2017). NMR and DFT investigations of structure of colchicine in various solvents including density functional theory calculations. Sci. Rep..

[41] Touw SI, de Groot HJ, Buda F (2004). DFT calculations of the 1H chemical shifts and 13C chemical shift tensors of retinal isomers. J. Mol. Struct. (Thoechem).

[42] Ditchfield R (1974). Self-consistent perturbation theory of diamagnetism: I. A gauge-invariant LCAO method for NMR chemical shifts. Mol. Phys..

[43] Becke AD (1993). A new mixing of Hartree-Fock and local density-functional theories. J. Chem. Phys..

[44] Parr R, Yang W (1989). Density-Functional Theory of Atoms and Molecules.

[45] Zhao Y, Truhlar DG (2008). The M06 suite of density functionals for main group thermochemistry, thermochemical kinetics, noncovalent interactions, excited states, and transition elements: two new functionals and systematic testing of four M06-class functionals and 12 other functionals. Theoret. Chem. Acc..

[46] Frisch MJ, Pople JA, Binkley JS (1984). Self-consistent molecular orbital methods 25. Supplementary functions for Gaussian basis sets. J. Chem. Phys..

[47] Weigend F, Ahlrichs R (2005). Balanced basis sets of split valence, triple zeta valence and quadruple zeta valence quality for H to Rn: Design and assessment of accuracy. Phys. Chem. Chem. Phys..

[48] Tomasi J, Mennucci B, Cammi R (2005). Quantum mechanical continuum solvation models. Chem. Rev..

[49] Gaussian09, R. A. 1, mj frisch, gw trucks, hb schlegel, ge scuseria, ma robb, jr cheeseman, g. Scalmani, v. Barone, b. Mennucci, ga petersson et al., gaussian. *Inc., Wallingford CT* **121**, 150–166 (2009).

[50] Chamkin AA (2021). Benchmarking DFT calculations of 1H and 13C chemical shifts in monosubstituted ferrocenes. Int. J. Quantum Chem..

