# Supplementary material for: DFT calculations of 1H- and 13C-NMR chemical shifts of 3-methyl-1-phenyl-4-(phenyldiazenyl)-1H-pyrazol-5-amine in solution
Source: Sci Rep. 2022 Oct 22;12:17798. doi: 10.1038/s41598-022-22900-y (PMC9588065; doi:10.1038/s41598-022-22900-y)
Supplement: Supplementary file 2 — Supplementary Information 2. [file 41598_2022_22900_MOESM2_ESM.docx]

**DFT Calculations of ^1^H- and ^13^C-NMR Chemical Shifts of 3-methyl-1-phenyl-4-(phenyldiazenyl)-1H-pyrazol-5-amine in Solution**

Zaki S. Safi^a*^ and Nuha Wazzan^b^

**^a^** Department of Chemistry, Faculty of Science, Al Azhar University-Gaza, P.O. Box 1277, Gaza, Palestine

**^b^** King Abdulaziz University, Chemistry Department, Faculty of Science, P.O. Box 4280, Jeddah 21589, Saudi Arabia.

***Corresponding Author:** [**zaki.safi@gamil.com**](mailto:zaki.safi@gamil.com)**;** [**z.safi@alazhar.edu.ps**](mailto:z.safi@alazhar.edu.ps)

**Supplementary materials**

**First: Supplementary Tables**

Table S1: Computed $\sigma_{TMS}$ for ^1^H-NMR and ^13^C-NMR chemical shifts of TMS reference.

|  | **H** | **C** |  | **H** | **C** |  | **H** | **C** |  | **H** | **C** |
| --- | --- | --- | --- | --- | --- | --- | --- | --- | --- | --- | --- |
|  | **C1** | |  | **C2** | |  | **C3** | |  | **C4** | |
| M6-2X | 32.067 | 188.3 |  | 32.035 | 187.5 |  | 32.025 | 188.4 |  | 31.995 | 187.5 |
| B3LYP | 31.999 | 185.2 |  | 31.988 | 183.9 |  | 31.961 | 185.2 |  | 31.947 | 183.8 |
| BP86 | 31.841 | 185.8 |  | 31.823 | 184.5 |  | 31.803 | 185.8 |  | 31.783 | 184.4 |
| B3PW91 | 31.897 | 188.2 |  | 31.875 | 186.9 |  | 31.859 | 188.2 |  | 31.835 | 186.8 |
| B97D | 32.005 | 185.5 |  | 31.982 | 184.1 |  | 31.969 | 185.5 |  | 31.943 | 184.0 |
| CAM-B3LYP | 31.950 | 188.3 |  | 31.937 | 187.0 |  | 31.911 | 188.2 |  | 31.896 | 186.9 |
| WB97XD | 31.971 | 189.8 |  | 31.947 | 188.8 |  | 31.931 | 189.7 |  | 31.906 | 188.6 |
| HSEH1PBE | 31.841 | 189.2 |  | 31.820 | 187.9 |  | 31.802 | 189.1 |  | 31.779 | 187.9 |
| LC-WPBE | 31.908 | 194.3 |  | 31.881 | 192.8 |  | 31.866 | 194.1 |  | 31.839 | 192.7 |
| LSDA | 31.367 | 185.6 |  | 31.356 | 183.9 |  | 31.329 | 185.5 |  | 31.314 | 183.8 |
| PBE1PBE | 31.834 | 189.8 |  | 31.810 | 188.6 |  | 31.795 | 189.7 |  | 31.770 | 188.5 |
| PW91PW91 | 31.795 | 185.6 |  | 31.775 | 183.8 |  | 31.758 | 185.6 |  | 31.734 | 183.8 |
| TPSSTPSS | 32.060 | 187.6 |  | 32.036 | 186.1 |  | 32.021 | 187.5 |  | 31.995 | 186.0 |
|  | C5 | |  | C6 | |  | C7 | |  | C8 | |
| M6-2X | 32.067 | 188.3 |  | 32.035 | 187.5 |  | 32.026 | 188.4 |  | 31.996 | 187.5 |
| B3LYP | 31.999 | 185.2 |  | 31.988 | 183.9 |  | 31.961 | 185.2 |  | 31.948 | 183.8 |
| BP86 | 31.841 | 185.8 |  | 31.823 | 184.5 |  | 31.803 | 185.8 |  | 31.783 | 184.4 |
| B3PW91 | 31.897 | 188.2 |  | 31.875 | 186.9 |  | 31.858 | 188.2 |  | 31.835 | 186.8 |
| B97D | 32.005 | 185.5 |  | 31.982 | 184.1 |  | 31.968 | 185.5 |  | 31.943 | 184.0 |
| CAM-B3LYP | 31.950 | 188.3 |  | 31.937 | 187.0 |  | 31.910 | 188.2 |  | 31.896 | 186.9 |
| WB97XD | 31.971 | 189.8 |  | 31.947 | 188.8 |  | 31.931 | 189.7 |  | 31.906 | 188.6 |
| HSEH1PBE | 31.908 | 194.3 |  | 31.820 | 187.9 |  | 31.802 | 189.1 |  | 31.779 | 187.9 |
| LC-WPBE | 31.908 | 194.3 |  | 31.881 | 192.8 |  | 31.866 | 194.1 |  | 31.839 | 192.7 |
| LSDA | 31.367 | 185.6 |  | 31.356 | 183.9 |  | 31.328 | 185.5 |  | 31.314 | 183.8 |
| PBE1PBE | 31.834 | 189.8 |  | 31.810 | 188.6 |  | 31.795 | 189.7 |  | 31.769 | 188.5 |
| PW91PW91 | 31.795 | 185.6 |  | 31.775 | 183.8 |  | 31.757 | 185.6 |  | 31.734 | 183.8 |
| TPSSTPSS | 32.060 | 187.6 |  | 32.036 | 186.1 |  | 32.021 | 187.5 |  | 31.995 | 186.0 |

Table S2. The average computed ^1^H NMR chemical shifts.

|  | **c7** | **c8** | **c9** | **c10** | **c12** | **c13** | **C14** |  | **c7** | **c8** | **c9** | **c10** | **c12** | **c13** | **C14** |
| --- | --- | --- | --- | --- | --- | --- | --- | --- | --- | --- | --- | --- | --- | --- | --- |
| Exp. | 7.54 | 8.4 | 7.37 | 2.55 | 7.72 | 7.43 | 7.31 |  | 7.54 | 8.4 | 7.37 | 2.55 | 7.72 | 7.43 | 7.31 |
|  | **group 1** | | | | | | |  | **group 1** | | | | | | |
| M6-2X | 8.4 | 8.45 | 8.24 | 2.61 | 8.88 | 8.37 | 8.01 |  | 8.44 | 8.57 | 8.42 | 2.65 | 8.73 | 8.42 | 8.34 |
| B3LYP | 7.76 | 7.81 | 7.70 | 2.48 | 8.22 | 7.74 | 7.48 |  | 7.86 | 7.90 | 7.80 | 2.44 | 8.28 | 7.82 | 7.56 |
| BP86 | 7.70 | 7.77 | 7.67 | 2.4 | 8.14 | 7.70 | 7.45 |  | 7.83 | 7.87 | 7.77 | 2.38 | 8.23 | 7.79 | 7.55 |
| B3PW91 | 7.80 | 7.86 | 7.77 | 2.45 | 8.24 | 7.79 | 7.54 |  | 7.92 | 7.96 | 7.86 | 2.43 | 8.31 | 7.88 | 7.63 |
| B97D | 7.63 | 7.68 | 7.57 | 2.42 | 8.07 | 7.61 | 7.35 |  | 7.72 | 7.78 | 7.69 | 2.39 | 8.13 | 7.72 | 7.48 |
| CAM-B3LYP | 7.87 | 7.90 | 7.79 | 2.47 | 8.33 | 7.82 | 7.55 |  | 7.99 | 8.01 | 7.90 | 2.48 | 8.41 | 7.94 | 7.67 |
| WB97XD | 7.89 | 7.97 | 7.90 | 2.47 | 8.31 | 7.93 | 7.68 |  | 8.03 | 8.00 | 7.83 | 2.47 | 8.42 | 7.89 | 7.61 |
| HSEH1PBE | 7.83 | 7.89 | 7.80 | 2.46 | 8.28 | 7.83 | 7.58 |  | 7.95 | 7.98 | 7.87 | 2.43 | 8.36 | 7.90 | 7.64 |
| LC-WPBE | 8.03 | 8.09 | 7.99 | 2.49 | 8.43 | 8.02 | 7.77 |  | 8.14 | 8.15 | 8.05 | 2.48 | 8.51 | 8.08 | 7.82 |
| LSDA | 7.83 | 7.91 | 7.83 | 2.40 | 8.28 | 7.86 | 7.61 |  | 7.96 | 8.01 | 7.92 | 2.36 | 8.37 | 7.94 | 7.71 |
| PBE1PBE | 7.84 | 7.90 | 7.8 | 2.46 | 8.29 | 7.83 | 7.57 |  | 7.96 | 8.00 | 7.90 | 2.44 | 8.35 | 7.93 | 7.68 |
| PW91PW91 | 7.70 | 7.77 | 7.68 | 2.39 | 8.15 | 7.71 | 7.45 |  | 7.80 | 7.86 | 7.77 | 2.37 | 8.22 | 7.81 | 7.56 |
| TPSSTPSS | 7.62 | 7.68 | 7.58 | 2.42 | 8.08 | 7.61 | 7.34 |  | 7.72 | 7.78 | 7.68 | 2.4 | 8.12 | 7.71 | 7.47 |
|  | **group 3** | | | | | | |  | **group 4** | | | | | | |
| M6-2X | 8.36 | 8.41 | 8.21 | 2.59 | 8.84 | 8.33 | 7.98 |  | 8.43 | 8.56 | 8.41 | 2.65 | 8.71 | 8.41 | 8.32 |
| B3LYP | 7.73 | 7.78 | 7.68 | 2.45 | 8.19 | 7.7 | 7.44 |  | 7.86 | 7.89 | 7.79 | 2.44 | 8.27 | 7.8 | 7.55 |
| BP86 | 7.67 | 7.74 | 7.64 | 2.38 | 8.11 | 7.67 | 7.42 |  | 7.82 | 7.86 | 7.77 | 2.37 | 8.21 | 7.78 | 7.53 |
| B3PW91 | 7.73 | 7.78 | 7.69 | 2.38 | 8.15 | 7.71 | 7.46 |  | 7.91 | 7.95 | 7.85 | 2.43 | 8.29 | 7.86 | 7.62 |
| B97D | 7.6 | 7.65 | 7.55 | 2.40 | 8.04 | 7.58 | 7.32 |  | 7.72 | 7.77 | 7.68 | 2.39 | 8.11 | 7.71 | 7.47 |
| CAM-B3LYP | 7.86 | 7.91 | 7.8 | 2.48 | 8.31 | 7.83 | 7.56 |  | 7.98 | 8.00 | 7.89 | 2.47 | 8.39 | 7.92 | 7.66 |
| WB97XD | 7.86 | 7.94 | 7.87 | 2.45 | 8.28 | 7.89 | 7.65 |  | 8.02 | 7.99 | 7.82 | 2.47 | 8.4 | 7.88 | 7.59 |
| HSEH1PBE | 7.80 | 7.87 | 7.77 | 2.44 | 8.24 | 7.80 | 7.54 |  | 7.94 | 7.97 | 7.86 | 2.43 | 8.34 | 7.88 | 7.62 |
| LC-WPBE | 8.01 | 8.06 | 7.96 | 2.46 | 8.39 | 7.99 | 7.73 |  | 8.13 | 8.14 | 8.03 | 2.48 | 8.49 | 8.07 | 7.81 |
| LSDA | 7.80 | 7.89 | 7.80 | 2.37 | 8.24 | 7.83 | 7.58 |  | 7.95 | 8.00 | 7.91 | 2.36 | 8.35 | 7.93 | 7.69 |
| PBE1PBE | 7.82 | 7.87 | 7.77 | 2.44 | 8.25 | 7.8 | 7.53 |  | 7.95 | 7.98 | 7.89 | 2.44 | 8.34 | 7.91 | 7.66 |
| PW91PW91 | 7.68 | 7.75 | 7.65 | 2.37 | 8.12 | 7.68 | 7.42 |  | 7.79 | 7.85 | 7.76 | 2.37 | 8.21 | 7.80 | 7.55 |
| TPSSTPSS | 7.60 | 7.65 | 7.55 | 2.39 | 8.04 | 7.57 | 7.31 |  | 7.72 | 7.76 | 7.67 | 2.40 | 8.11 | 7.69 | 7.46 |
|  | **group 5** | | | | | | |  | **group 6** | | | | | | |
| M6-2X | 8.41 | 8.43 | 8.23 | 2.59 | 8.86 | 8.35 | 8.02 |  | 8.45 | 8.55 | 8.41 | 2.62 | 8.74 | 8.37 | 8.31 |
| B3LYP | 7.79 | 7.81 | 7.69 | 2.45 | 8.24 | 7.71 | 7.45 |  | 7.88 | 7.89 | 7.78 | 2.42 | 8.3 | 7.78 | 7.54 |
| BP86 | 7.73 | 7.77 | 7.66 | 2.38 | 8.17 | 7.67 | 7.42 |  | 7.84 | 7.87 | 7.76 | 2.35 | 8.25 | 7.76 | 7.53 |
| B3PW91 | 7.83 | 7.86 | 7.76 | 2.43 | 8.26 | 7.76 | 7.51 |  | 7.94 | 7.95 | 7.84 | 2.41 | 8.33 | 7.85 | 7.61 |
| B97D | 7.66 | 7.68 | 7.57 | 2.4 | 8.09 | 7.58 | 7.33 |  | 7.74 | 7.77 | 7.68 | 2.37 | 8.15 | 7.69 | 7.46 |
| CAM-B3LYP | 7.92 | 7.93 | 7.82 | 2.48 | 8.36 | 7.83 | 7.56 |  | 8.00 | 8.00 | 7.89 | 2.45 | 8.42 | 7.90 | 7.65 |
| WB97XD | 7.92 | 7.97 | 7.88 | 2.45 | 8.33 | 7.89 | 7.66 |  | 8.05 | 8.00 | 7.83 | 2.45 | 8.44 | 7.85 | 7.58 |
| HSEH1PBE | 7.89 | 7.93 | 7.82 | 2.47 | 8.33 | 7.83 | 7.58 |  | 7.96 | 7.97 | 7.86 | 2.41 | 8.38 | 7.86 | 7.61 |
| LC-WPBE | 8.06 | 8.09 | 7.98 | 2.46 | 8.45 | 7.99 | 7.74 |  | 8.15 | 8.14 | 8.03 | 2.45 | 8.53 | 8.05 | 7.8 |
| LSDA | 7.86 | 7.92 | 7.82 | 2.37 | 8.30 | 7.83 | 7.59 |  | 7.97 | 8.01 | 7.91 | 2.34 | 8.39 | 7.91 | 7.69 |
| PBE1PBE | 7.87 | 7.90 | 7.79 | 2.44 | 8.30 | 7.80 | 7.54 |  | 7.97 | 7.99 | 7.88 | 2.42 | 8.38 | 7.90 | 7.65 |
| PW91PW91 | 7.73 | 7.78 | 7.67 | 2.37 | 8.18 | 7.68 | 7.43 |  | 7.82 | 7.85 | 7.76 | 2.35 | 8.25 | 7.78 | 7.55 |
| TPSSTPSS | 7.65 | 7.68 | 7.57 | 2.39 | 8.10 | 7.58 | 7.32 |  | 7.74 | 7.77 | 7.67 | 2.37 | 8.15 | 7.68 | 7.45 |
|  | **group 7** | | | | | | |  | **group 8** | | | | | | |
| M6-2X | 8.37 | 8.4 | 8.19 | 2.56 | 8.82 | 8.31 | 8 |  | 8.43 | 8.54 | 8.4 | 2.62 | 8.72 | 8.35 | 8.3 |
| B3LYP | 7.76 | 7.78 | 7.66 | 2.43 | 8.2 | 7.67 | 7.42 |  | 7.87 | 7.88 | 7.77 | 2.41 | 8.27 | 7.77 | 7.53 |
| BP86 | 7.69 | 7.74 | 7.63 | 2.35 | 8.12 | 7.64 | 7.39 |  | 7.83 | 7.85 | 7.75 | 2.34 | 8.22 | 7.75 | 7.51 |
| B3PW91 | 7.80 | 7.83 | 7.72 | 2.40 | 8.21 | 7.73 | 7.48 |  | 7.92 | 7.94 | 7.83 | 2.40 | 8.30 | 7.83 | 7.6 |
| B97D | 7.63 | 7.65 | 7.54 | 2.37 | 8.05 | 7.55 | 7.29 |  | 7.73 | 7.76 | 7.67 | 2.36 | 8.12 | 7.68 | 7.45 |
| CAM-B3LYP | 7.88 | 7.9 | 7.79 | 2.45 | 8.32 | 7.79 | 7.53 |  | 7.99 | 7.99 | 7.88 | 2.44 | 8.4 | 7.89 | 7.63 |
| WB97XD | 7.88 | 7.94 | 7.85 | 2.42 | 8.29 | 7.85 | 7.62 |  | 8.04 | 7.98 | 7.81 | 2.44 | 8.42 | 7.84 | 7.56 |
| HSEH1PBE | 7.83 | 7.86 | 7.76 | 2.41 | 8.25 | 7.76 | 7.52 |  | 7.95 | 7.96 | 7.84 | 2.40 | 8.35 | 7.85 | 7.6 |
| LC-WPBE | 8.03 | 8.06 | 7.95 | 2.43 | 8.4 | 7.95 | 7.7 |  | 8.14 | 8.13 | 8.02 | 2.44 | 8.50 | 8.03 | 7.78 |
| LSDA | 7.82 | 7.88 | 7.79 | 2.35 | 8.25 | 7.79 | 7.56 |  | 7.96 | 7.99 | 7.90 | 2.33 | 8.37 | 7.9 | 7.68 |
| PBE1PBE | 7.84 | 7.87 | 7.76 | 2.41 | 8.26 | 7.76 | 7.51 |  | 7.96 | 7.97 | 7.87 | 2.41 | 8.35 | 7.88 | 7.64 |
| PW91PW91 | 7.70 | 7.75 | 7.64 | 2.35 | 8.13 | 7.65 | 7.39 |  | 7.81 | 7.84 | 7.74 | 2.34 | 8.22 | 7.77 | 7.53 |
| TPSSTPSS | 7.62 | 7.65 | 7.54 | 2.37 | 8.06 | 7.54 | 7.28 |  | 7.72 | 7.75 | 7.65 | 2.36 | 8.12 | 7.66 | 7.44 |

Table S3. The average computed ^13^C NMR chemical shifts.

|  | c3 | c4 | c5 | C6 | c7 | c8 | c9 | c10 | C11 | c12 | c13 | c14 |
| --- | --- | --- | --- | --- | --- | --- | --- | --- | --- | --- | --- | --- |
| Exp. | 150.1 | 123.1 | 137.7 | 137.4 | 123.4 | 129.7 | 127.8 | 11.4 | 153.3 | 121.1 | 128.9 | 128.3 |
| **C1** |  |  |  |  |  |  |  |  |  |  |  |  |
| M6-2X | 174.7 | 145.8 | 163.9 | 161.6 | 145.1 | 152.3 | 149.9 | 14.0 | 170.4 | 133.5 | 151.7 | 148.6 |
| B3LYP | 158.0 | 134.2 | 152.2 | 147.0 | 128.9 | 135.8 | 132.5 | 13.5 | 156.3 | 118.9 | 135.5 | 132.2 |
| BP86 | 152.1 | 132.9 | 146.3 | 143.6 | 125.7 | 132.8 | 129.4 | 13.4 | 152.6 | 116.0 | 132.4 | 129.5 |
| B3PW91 | 155.9 | 132.6 | 149.9 | 144.8 | 127.5 | 134.5 | 131.2 | 12.4 | 153.8 | 117.6 | 134.0 | 130.9 |
| B97D | 149.9 | 130.6 | 144.5 | 140.9 | 122.9 | 129.6 | 126.2 | 13.8 | 149.9 | 113.5 | 129.3 | 126.3 |
| CAM-B3LYP | 161.7 | 133.7 | 155.8 | 149.1 | 131.6 | 138.6 | 135.3 | 13.0 | 158.2 | 121.2 | 138.2 | 134.6 |
| WB97XD | 160.3 | 133.0 | 154.2 | 148.1 | 130.4 | 137.4 | 134.5 | 13.4 | 156.4 | 120.1 | 137.0 | 133.5 |
| HSEH1PBE | 157.7 | 133.7 | 151.7 | 146.4 | 129.2 | 136.3 | 132.9 | 13.2 | 155.5 | 119.2 | 135.8 | 132.6 |
| LC-WPBE | 165.0 | 133.7 | 158.6 | 150.8 | 134.7 | 141.6 | 138.6 | 13.6 | 159.0 | 124.4 | 141.1 | 137.6 |
| LSDA | 157.0 | 137.3 | 149.7 | 148.0 | 130.6 | 138.2 | 134.9 | 12.0 | 157.3 | 120.6 | 137.8 | 135.2 |
| PBE1PBE | 157.4 | 133.2 | 151.5 | 146.0 | 129.0 | 136.1 | 132.8 | 13.3 | 155.0 | 119.0 | 135.6 | 132.4 |
| PW91PW91 | 152.6 | 133.4 | 146.8 | 144.3 | 126.4 | 133.5 | 130.2 | 13.2 | 153.3 | 116.6 | 133.2 | 130.3 |
| TPSSTPSS | 149.5 | 129.7 | 145.2 | 140.9 | 122.7 | 129.4 | 126.1 | 14.3 | 149.4 | 113.6 | 129.1 | 126.3 |
| **group 2** |  |  |  |  |  |  |  |  |  |  |  |  |
| M6-2X | 174.2 | 145.0 | 162.0 | 158.6 | 142.9 | 150.8 | 148.7 | 14.4 | 168.8 | 130.3 | 150.3 | 147.2 |
| B3LYP | 158.6 | 134.2 | 151.1 | 145.5 | 128.6 | 135.4 | 132.1 | 14.0 | 155.7 | 118.8 | 135.3 | 131.8 |
| BP86 | 152.7 | 133.0 | 145.1 | 141.8 | 125.4 | 132.4 | 129.1 | 14.0 | 152.0 | 116.0 | 132.4 | 129.2 |
| B3PW91 | 157.0 | 133.4 | 149.4 | 144.0 | 127.9 | 134.8 | 131.5 | 14.1 | 154.0 | 118.3 | 134.6 | 131.2 |
| B97D | 150.0 | 130.1 | 142.6 | 138.4 | 122.0 | 128.5 | 125.3 | 14.3 | 148.5 | 112.9 | 128.6 | 125.4 |
| CAM-B3LYP | 162.9 | 134.0 | 155.5 | 148.6 | 131.6 | 138.5 | 135.3 | 13.7 | 158.2 | 121.4 | 138.3 | 134.5 |
| WB97XD | 159.8 | 131.8 | 152.4 | 145.2 | 129.3 | 136.3 | 133.2 | 14.0 | 154.7 | 119.2 | 135.9 | 132.1 |
| HSEH1PBE | 157.6 | 133.1 | 149.9 | 144.2 | 128.2 | 135.2 | 131.8 | 13.7 | 154.3 | 118.6 | 135.0 | 131.6 |
| LC-WPBE | 164.2 | 132.4 | 156.7 | 149.2 | 133.3 | 140.5 | 137.2 | 14.0 | 157.9 | 123.3 | 140.1 | 136.1 |
| LSDA | 158.4 | 138.1 | 149.2 | 146.7 | 130.9 | 138.5 | 135.2 | 12.6 | 157.5 | 121.2 | 138.4 | 135.4 |
| PBE1PBE | 157.2 | 132.7 | 149.7 | 144.0 | 128.0 | 135.0 | 131.7 | 13.8 | 153.9 | 118.3 | 134.8 | 131.3 |
| PW91PW91 | 153.4 | 133.4 | 145.4 | 142.2 | 125.9 | 132.8 | 129.5 | 13.6 | 152.6 | 116.5 | 132.9 | 129.7 |
| TPSSTPSS | 149.7 | 129.6 | 143.6 | 138.7 | 122.0 | 128.7 | 125.5 | 14.9 | 148.5 | 113.1 | 128.7 | 125.6 |
| **group 3** |  |  |  |  |  |  |  |  |  |  |  |  |
| M6-2X | 174.8 | 146.1 | 164.1 | 161.5 | 145.1 | 152.3 | 149.9 | 14.2 | 170.4 | 133.5 | 151.7 | 148.7 |
| B3LYP | 158.1 | 134.3 | 152.3 | 146.8 | 128.8 | 135.7 | 132.4 | 13.5 | 156.2 | 118.8 | 135.4 | 132.2 |
| BP86 | 152.2 | 133.0 | 146.4 | 143.5 | 125.6 | 132.7 | 129.3 | 13.5 | 152.5 | 115.9 | 132.4 | 129.5 |
| B3PW91 | 155.8 | 132.6 | 149.9 | 144.5 | 127.2 | 134.2 | 131.0 | 12.3 | 153.6 | 117.5 | 133.8 | 130.8 |
| B97D | 150.0 | 130.8 | 144.6 | 140.8 | 122.8 | 129.5 | 126.2 | 13.9 | 149.8 | 113.5 | 129.3 | 126.3 |
| CAM-B3LYP | 162.6 | 134.3 | 156.6 | 149.4 | 132.0 | 138.8 | 135.7 | 13.1 | 158.5 | 121.6 | 138.4 | 135.0 |
| WB97XD | 160.3 | 133.1 | 154.2 | 147.8 | 130.2 | 137.2 | 134.3 | 13.3 | 156.2 | 119.9 | 136.8 | 133.4 |
| HSEH1PBE | 157.7 | 133.9 | 151.8 | 146.2 | 129.0 | 136.2 | 132.8 | 13.3 | 155.4 | 119.1 | 135.8 | 132.6 |
| LC-WPBE | 164.9 | 133.7 | 158.6 | 150.6 | 134.5 | 141.4 | 138.4 | 13.6 | 158.8 | 124.2 | 141.0 | 137.5 |
| LSDA | 157.1 | 137.4 | 149.8 | 147.9 | 130.5 | 138.1 | 134.8 | 12.0 | 157.2 | 120.5 | 137.7 | 135.2 |
| PBE1PBE | 157.4 | 133.2 | 151.5 | 145.9 | 128.9 | 136.0 | 132.7 | 13.3 | 154.9 | 118.9 | 135.5 | 132.4 |
| PW91PW91 | 152.7 | 133.5 | 146.9 | 144.2 | 126.3 | 133.5 | 130.1 | 13.3 | 153.2 | 116.6 | 133.2 | 130.3 |
| TPSSTPSS | 149.6 | 129.9 | 145.3 | 140.8 | 122.6 | 129.3 | 126.1 | 14.4 | 149.3 | 113.6 | 129.1 | 126.3 |
| **group 4** |  |  |  |  |  |  |  |  |  |  |  |  |
| M6-2X | 174.3 | 145.2 | 162.2 | 158.4 | 142.9 | 150.7 | 148.6 | 14.5 | 168.7 | 130.2 | 150.3 | 147.3 |
| B3LYP | 158.6 | 134.3 | 151.2 | 145.3 | 128.5 | 135.3 | 132.0 | 14.1 | 155.6 | 118.8 | 135.3 | 131.8 |
| BP86 | 152.7 | 133.1 | 145.2 | 141.6 | 125.3 | 132.3 | 129.0 | 14.1 | 151.9 | 115.9 | 132.4 | 129.2 |
| B3PW91 | 157.1 | 133.5 | 149.5 | 143.8 | 127.8 | 134.8 | 131.4 | 14.2 | 153.9 | 118.3 | 134.6 | 131.3 |
| B97D | 150.0 | 130.3 | 142.7 | 138.2 | 121.9 | 128.5 | 125.3 | 14.4 | 148.4 | 112.8 | 128.6 | 125.4 |
| CAM-B3LYP | 162.9 | 134.1 | 155.6 | 148.4 | 131.5 | 138.4 | 135.2 | 13.7 | 158.1 | 121.3 | 138.2 | 134.5 |
| WB97XD | 159.8 | 131.9 | 152.5 | 145.0 | 129.1 | 136.1 | 133.1 | 14.0 | 154.6 | 119.1 | 135.8 | 132.0 |
| HSEH1PBE | 157.6 | 133.3 | 150.0 | 144.0 | 128.1 | 135.1 | 131.8 | 13.7 | 154.2 | 118.5 | 135.0 | 131.6 |
| LC-WPBE | 164.3 | 132.6 | 156.8 | 149.0 | 133.1 | 140.3 | 137.1 | 14.0 | 157.8 | 123.2 | 140.0 | 136.1 |
| LSDA | 158.4 | 138.2 | 149.3 | 146.5 | 130.8 | 138.4 | 135.1 | 12.7 | 157.4 | 121.1 | 138.3 | 135.4 |
| PBE1PBE | 157.3 | 132.8 | 149.8 | 143.8 | 127.8 | 134.9 | 131.6 | 13.8 | 153.7 | 118.2 | 134.7 | 131.3 |
| PW91PW91 | 153.4 | 133.5 | 145.5 | 142.0 | 125.8 | 132.7 | 129.5 | 13.7 | 152.5 | 116.4 | 132.8 | 129.7 |
| TPSSTPSS | 149.8 | 129.8 | 143.7 | 138.5 | 122.0 | 128.6 | 125.4 | 14.9 | 148.4 | 113.1 | 128.6 | 125.6 |
| **group 5** |  |  |  |  |  |  |  |  |  |  |  |  |
| M6-2X | 172.5 | 144.5 | 161.9 | 160.6 | 144.0 | 151.5 | 148.8 | 13.6 | 169.6 | 132.8 | 151.2 | 147.9 |
| B3LYP | 156.4 | 133.1 | 150.6 | 146.1 | 128.0 | 135.2 | 131.6 | 13.3 | 155.6 | 118.4 | 134.9 | 131.7 |
| BP86 | 150.7 | 131.9 | 144.8 | 142.8 | 124.8 | 132.2 | 128.6 | 13.2 | 152.0 | 115.5 | 132.0 | 129.1 |
| B3PW91 | 154.2 | 131.6 | 148.3 | 144.0 | 126.6 | 133.9 | 130.4 | 12.2 | 153.1 | 117.1 | 133.5 | 130.4 |
| B97D | 148.5 | 129.7 | 143.1 | 140.2 | 122.0 | 129.0 | 125.5 | 13.7 | 149.3 | 113.0 | 128.9 | 125.9 |
| CAM-B3LYP | 160.7 | 133.2 | 154.8 | 148.8 | 131.2 | 138.4 | 135.0 | 13.0 | 157.9 | 121.1 | 138.0 | 134.5 |
| WB97XD | 158.5 | 132.0 | 152.5 | 147.3 | 129.5 | 136.8 | 133.6 | 13.2 | 155.7 | 119.6 | 136.5 | 133.0 |
| HSEH1PBE | 158.6 | 135.2 | 152.6 | 148.1 | 130.8 | 138.2 | 134.7 | 15.6 | 157.4 | 121.2 | 137.9 | 134.7 |
| LC-WPBE | 163.0 | 132.7 | 156.8 | 150.0 | 133.7 | 141.1 | 137.7 | 13.4 | 158.3 | 123.8 | 140.6 | 137.1 |
| LSDA | 155.6 | 136.3 | 148.2 | 147.3 | 129.7 | 137.7 | 134.2 | 11.8 | 156.8 | 120.0 | 137.3 | 134.7 |
| PBE1PBE | 155.8 | 132.2 | 149.9 | 145.2 | 128.1 | 135.5 | 132.0 | 13.1 | 154.3 | 118.5 | 135.1 | 131.9 |
| PW91PW91 | 151.2 | 132.4 | 145.3 | 143.5 | 125.5 | 133.0 | 129.4 | 13.0 | 152.7 | 116.1 | 132.7 | 129.8 |
| TPSSTPSS | 148.1 | 128.8 | 143.7 | 140.1 | 121.8 | 128.9 | 125.4 | 14.1 | 148.8 | 113.1 | 128.6 | 125.8 |
| **group 6** |  |  |  |  |  |  |  |  |  |  |  |  |
| M6-2X | 172.2 | 144.0 | 160.1 | 157.4 | 141.8 | 150.1 | 147.7 | 14.2 | 167.9 | 129.9 | 149.8 | 146.4 |
| B3LYP | 156.9 | 133.2 | 149.4 | 144.6 | 127.7 | 134.8 | 131.3 | 13.9 | 155.0 | 118.2 | 134.8 | 131.3 |
| BP86 | 151.3 | 132.0 | 143.6 | 140.8 | 124.5 | 131.9 | 128.3 | 13.9 | 151.4 | 115.3 | 131.9 | 128.8 |
| B3PW91 | 154.9 | 131.8 | 147.3 | 142.6 | 126.5 | 133.8 | 130.2 | 13.4 | 152.8 | 117.2 | 133.6 | 130.3 |
| B97D | 148.6 | 129.2 | 141.1 | 137.5 | 121.1 | 128.0 | 124.6 | 14.2 | 147.8 | 112.2 | 128.2 | 125.0 |
| CAM-B3LYP | 161.0 | 133.0 | 153.7 | 147.7 | 130.7 | 137.9 | 134.4 | 13.5 | 157.5 | 120.8 | 137.8 | 134.0 |
| WB97XD | 158.0 | 130.8 | 150.7 | 144.3 | 128.3 | 135.7 | 132.3 | 13.8 | 154.0 | 118.6 | 135.4 | 131.5 |
| HSEH1PBE | 155.9 | 132.1 | 148.3 | 143.3 | 127.3 | 134.7 | 131.1 | 13.5 | 153.7 | 118.0 | 134.6 | 131.1 |
| LC-WPBE | 162.4 | 131.5 | 155.0 | 148.4 | 132.3 | 139.9 | 136.4 | 13.8 | 157.2 | 122.7 | 139.6 | 135.6 |
| LSDA | 156.9 | 137.2 | 147.7 | 145.8 | 130.0 | 137.9 | 134.5 | 12.5 | 156.9 | 120.6 | 137.9 | 135.0 |
| PBE1PBE | 155.6 | 131.7 | 148.0 | 143.1 | 127.1 | 134.4 | 130.9 | 13.6 | 153.2 | 117.7 | 134.3 | 130.9 |
| PW91PW91 | 151.9 | 132.4 | 143.9 | 141.3 | 125.1 | 132.3 | 128.8 | 13.5 | 152.0 | 115.8 | 132.4 | 129.3 |
| TPSSTPSS | 148.3 | 128.7 | 142.1 | 137.7 | 121.2 | 128.2 | 124.8 | 14.7 | 147.9 | 112.5 | 128.2 | 125.2 |
| **group 7** |  |  |  |  |  |  |  |  |  |  |  |  |
| M6-2X | 172.8 | 144.9 | 162.2 | 160.6 | 144.1 | 151.5 | 148.9 | 13.8 | 169.6 | 132.8 | 151.3 | 148.0 |
| B3LYP | 156.5 | 133.4 | 150.7 | 146.0 | 128.0 | 135.1 | 131.6 | 13.3 | 155.5 | 118.3 | 134.9 | 131.6 |
| BP86 | 150.8 | 132.2 | 145.0 | 142.8 | 124.8 | 132.1 | 128.6 | 13.2 | 152.0 | 115.4 | 131.9 | 129.1 |
| B3PW91 | 154.4 | 131.9 | 148.5 | 143.9 | 126.5 | 133.8 | 130.3 | 12.2 | 153.1 | 117.0 | 133.5 | 130.4 |
| B97D | 148.7 | 130.0 | 143.3 | 140.1 | 122.0 | 129.0 | 125.5 | 13.7 | 149.3 | 112.9 | 128.8 | 125.9 |
| CAM-B3LYP | 160.8 | 133.4 | 154.9 | 148.6 | 131.1 | 138.2 | 134.8 | 12.9 | 157.8 | 121.0 | 137.9 | 134.4 |
| WB97XD | 158.5 | 132.2 | 152.5 | 147.0 | 129.3 | 136.6 | 133.5 | 13.1 | 155.6 | 119.4 | 136.3 | 132.8 |
| HSEH1PBE | 156.2 | 133.0 | 150.3 | 145.5 | 128.2 | 135.6 | 132.0 | 13.0 | 154.8 | 118.5 | 135.2 | 132.1 |
| LC-WPBE | 163.1 | 132.9 | 156.9 | 149.8 | 133.5 | 140.9 | 137.6 | 13.3 | 158.1 | 123.6 | 140.4 | 137.0 |
| LSDA | 155.8 | 136.6 | 148.4 | 147.2 | 129.7 | 137.6 | 134.1 | 11.8 | 156.7 | 120.0 | 137.2 | 134.7 |
| PBE1PBE | 155.9 | 132.5 | 150.0 | 145.1 | 128.0 | 135.4 | 131.9 | 13.1 | 154.2 | 118.4 | 135.0 | 131.9 |
| PW91PW91 | 151.4 | 132.7 | 145.6 | 143.5 | 125.5 | 132.9 | 129.4 | 13.0 | 152.7 | 116.1 | 132.7 | 129.8 |
| TPSSTPSS | 148.3 | 129.1 | 144.0 | 140.1 | 121.8 | 128.8 | 125.3 | 14.1 | 148.8 | 113.0 | 128.6 | 125.8 |
| **group 8** |  |  |  |  |  |  |  |  |  |  |  |  |
| M6-2X | 172.4 | 144.3 | 160.4 | 157.4 | 141.8 | 150.0 | 147.6 | 14.3 | 167.9 | 129.9 | 149.8 | 146.4 |
| B3LYP | 157.1 | 133.4 | 149.6 | 144.4 | 127.6 | 134.7 | 131.2 | 13.9 | 155.0 | 118.1 | 134.7 | 131.3 |
| BP86 | 151.4 | 132.3 | 143.8 | 140.7 | 124.5 | 131.8 | 128.3 | 13.9 | 151.3 | 115.3 | 131.9 | 128.8 |
| B3PW91 | 155.1 | 132.1 | 147.5 | 142.4 | 126.4 | 133.7 | 130.2 | 13.4 | 152.7 | 117.1 | 133.6 | 130.3 |
| B97D | 148.7 | 129.5 | 141.3 | 137.4 | 121.1 | 127.9 | 124.6 | 14.2 | 147.8 | 112.1 | 128.2 | 125.0 |
| CAM-B3LYP | 161.2 | 133.3 | 153.8 | 147.5 | 130.6 | 137.8 | 134.4 | 13.5 | 157.4 | 120.7 | 137.7 | 134.0 |
| WB97XD | 158.2 | 131.1 | 150.9 | 144.2 | 128.2 | 135.6 | 132.3 | 13.8 | 153.9 | 118.5 | 135.3 | 131.5 |
| HSEH1PBE | 156.1 | 132.4 | 148.5 | 143.2 | 127.2 | 134.6 | 131.0 | 13.5 | 153.6 | 117.9 | 134.5 | 131.1 |
| LC-WPBE | 162.5 | 131.8 | 155.1 | 148.2 | 132.2 | 139.8 | 136.3 | 13.8 | 157.1 | 122.6 | 139.5 | 135.6 |
| LSDA | 157.1 | 137.4 | 147.9 | 145.6 | 129.9 | 137.8 | 134.4 | 12.5 | 156.8 | 120.5 | 137.9 | 135.0 |
| PBE1PBE | 155.8 | 132.0 | 148.2 | 143.0 | 127.0 | 134.4 | 130.8 | 13.6 | 153.1 | 117.6 | 134.2 | 130.9 |
| PW91PW91 | 152.1 | 132.7 | 144.1 | 141.1 | 125.0 | 132.2 | 128.8 | 13.5 | 151.9 | 115.8 | 132.4 | 129.3 |
| TPSSTPSS | 148.5 | 128.9 | 142.3 | 137.6 | 121.1 | 128.1 | 124.7 | 14.7 | 147.9 | 112.5 | 128.2 | 125.2 |

Table S4. Different combinations and abbreviations

|  | **NMR functional** | **G1** | **G2** | **G3** | **G4** | **G5** | **G6** | **G7** | **G8** |
| --- | --- | --- | --- | --- | --- | --- | --- | --- | --- |
| 1 | M6-2X | TBT | PBT | TBP | PBP | TMT | PMT | TMP | PMP |
| 2 | B3LYP |  |  |  |  |  |  |  |  |
| 3 | BP86 |  |  |  |  |  |  |  |  |
| 4 | B3PW91 |  |  |  |  |  |  |  |  |
| 5 | B97D |  |  |  |  |  |  |  |  |
| 6 | CAM-B3LYP |  |  |  |  |  |  |  |  |
| 7 | WB97XD |  |  |  |  |  |  |  |  |
| 8 | HSEH1PBE |  |  |  |  |  |  |  |  |
| 9 | LC-WPBE |  |  |  |  |  |  |  |  |
| 10 | LSDA |  |  |  |  |  |  |  |  |
| 11 | PBE1PBE |  |  |  |  |  |  |  |  |
| 12 | PW91PW91 |  |  |  |  |  |  |  |  |
| 13 | TPSSTPSS |  |  |  |  |  |  |  |  |

- 1^st^ letter corresponds to the basis set that used to compute the anisotropic chemical shifts T for TZVP) and P for 6-311+G(2d,p).
- 2^nd^ letter (B or M) corresponds to geometry functional (B for B3LYP and M for M06-2x).
- 3^rd^ letter corresponds to the basis set that is used to compute the anisotropic chemical shifts T for TZVP and P for 6-311+G(2d,p)

Table S5. Descriptors computed for ^1^H deviations for combinations of geometry, basis set, and NMR functional (C1-C4).

| Combination | **Functional** | **RMSE** | **MAX** | **MAE** | **MSE** | **ME** | **MAPE** | **R^2^** | **slope** | **intercept** |
| --- | --- | --- | --- | --- | --- | --- | --- | --- | --- | --- |
| TBT | B97D | 0.192 | 0.349 | 0.169 | 0.037 | 0.130 | 2.66% | 0.9979 | 1.065 | -0.310 |
|  | TPSSTPSS | 0.194 | 0.357 | 0.168 | 0.038 | 0.130 | 2.67% | 0.9976 | 1.065 | -0.311 |
|  | BP86 | 0.263 | 0.423 | 0.245 | 0.069 | 0.202 | 3.57% | 0.9981 | 1.086 | -0.381 |
|  | PW91PW91 | 0.270 | 0.433 | 0.252 | 0.073 | 0.207 | 3.66% | 0.9980 | 1.088 | -0.391 |
|  | B3LYP | 0.302 | 0.500 | 0.274 | 0.091 | 0.253 | 3.61% | 0.9977 | 1.080 | -0.290 |
|  | B3PW91 | 0.343 | 0.518 | 0.320 | 0.118 | 0.292 | 4.17% | 0.9982 | 1.094 | -0.344 |
|  | HSEH1PBE | 0.374 | 0.556 | 0.348 | 0.140 | 0.322 | 4.46% | 0.9982 | 1.100 | -0.352 |
|  | PBE1PBE | 0.379 | 0.565 | 0.352 | 0.144 | 0.326 | 4.51% | 0.9981 | 1.100 | -0.353 |
|  | CAM-B3LYP | 0.386 | 0.610 | 0.355 | 0.149 | 0.332 | 4.56% | 0.9975 | 1.101 | -0.349 |
|  | LSDA | 0.393 | 0.555 | 0.373 | 0.154 | 0.329 | 4.96% | 0.9984 | 1.116 | -0.456 |
|  | WB97XD | 0.442 | 0.592 | 0.413 | 0.196 | 0.391 | 5.08% | 0.9985 | 1.112 | -0.366 |
|  | LC-WPBE | 0.542 | 0.710 | 0.505 | 0.294 | 0.487 | 6.09% | 0.9988 | 1.131 | -0.403 |
|  | M6-2X | 0.855 | 1.159 | 0.792 | 0.731 | 0.792 | 9.44% | 0.9971 | 1.176 | -0.400 |
| PBT | TPSSTPSS | 0.267 | 0.403 | 0.253 | 0.072 | 0.210 | 3.62% | 0.9987 | 1.087 | 0.357 |
|  | B97D | 0.274 | 0.407 | 0.261 | 0.075 | 0.215 | 3.73% | 0.9987 | 1.090 | 0.371 |
|  | PW91PW91 | 0.351 | 0.505 | 0.336 | 0.123 | 0.285 | 4.65% | 0.9986 | 1.111 | 0.430 |
|  | BP86 | 0.351 | 0.505 | 0.336 | 0.123 | 0.286 | 4.66% | 0.9986 | 1.110 | 0.424 |
|  | B3LYP | 0.377 | 0.562 | 0.353 | 0.142 | 0.322 | 4.61% | 0.9983 | 1.104 | 0.358 |
|  | B3PW91 | 0.425 | 0.586 | 0.402 | 0.181 | 0.368 | 5.16% | 0.9988 | 1.117 | 0.385 |
|  | HSEH1PBE | 0.447 | 0.636 | 0.421 | 0.200 | 0.387 | 5.41% | 0.9984 | 1.122 | 0.400 |
|  | WB97XD | 0.464 | 0.697 | 0.427 | 0.215 | 0.406 | 5.39% | 0.9976 | 1.118 | 0.365 |
|  | PBE1PBE | 0.464 | 0.635 | 0.437 | 0.215 | 0.407 | 5.52% | 0.9987 | 1.123 | 0.390 |
|  | CAM-B3LYP | 0.480 | 0.686 | 0.446 | 0.230 | 0.425 | 5.51% | 0.9983 | 1.120 | 0.358 |
|  | LSDA | 0.484 | 0.650 | 0.464 | 0.234 | 0.410 | 6.12% | 0.9988 | 1.143 | 0.498 |
|  | LC-WPBE | 0.604 | 0.786 | 0.564 | 0.365 | 0.544 | 6.79% | 0.9989 | 1.147 | 0.399 |
|  | M6-2X | 0.937 | 1.083 | 0.879 | 0.877 | 0.879 | 10.13% | 0.9990 | 1.183 | 0.311 |
| TBP | B97D | 0.173 | 0.317 | 0.147 | 0.030 | 0.103 | 2.51% | 0.9979 | 1.063 | 0.320 |
|  | TPSSTPSS | 0.174 | 0.324 | 0.147 | 0.030 | 0.102 | 2.52% | 0.9976 | 1.063 | 0.324 |
|  | BP86 | 0.241 | 0.390 | 0.224 | 0.058 | 0.174 | 3.42% | 0.9982 | 1.084 | 0.378 |
|  | PW91PW91 | 0.248 | 0.400 | 0.230 | 0.061 | 0.180 | 3.51% | 0.9981 | 1.094 | 0.401 |
|  | B3PW91 | 0.277 | 0.434 | 0.261 | 0.077 | 0.213 | 3.81% | 0.9983 | 1.078 | 0.299 |
|  | B3LYP | 0.277 | 0.467 | 0.252 | 0.077 | 0.225 | 3.45% | 0.9977 | 1.092 | 0.388 |
|  | HSEH1PBE | 0.348 | 0.522 | 0.325 | 0.121 | 0.293 | 4.30% | 0.9982 | 1.098 | 0.348 |
|  | PBE1PBE | 0.352 | 0.531 | 0.329 | 0.124 | 0.297 | 4.35% | 0.9981 | 1.098 | 0.349 |
|  | LSDA | 0.368 | 0.522 | 0.351 | 0.135 | 0.301 | 4.81% | 0.9984 | 1.114 | 0.434 |
|  | CAM-B3LYP | 0.384 | 0.587 | 0.353 | 0.147 | 0.334 | 4.46% | 0.9979 | 1.097 | 0.309 |
|  | WB97XD | 0.414 | 0.557 | 0.389 | 0.172 | 0.361 | 4.91% | 0.9986 | 1.110 | 0.354 |
|  | LC-WPBE | 0.512 | 0.673 | 0.480 | 0.263 | 0.456 | 5.91% | 0.9988 | 1.129 | 0.381 |
|  | M6-2X | 0.824 | 1.123 | 0.760 | 0.679 | 0.760 | 8.99% | 0.9971 | 1.175 | 0.385 |
| PBP | TPSSTPSS | 0.257 | 0.387 | 0.243 | 0.066 | 0.199 | 3.53% | 0.9987 | 1.085 | 0.357 |
|  | B97D | 0.264 | 0.392 | 0.251 | 0.070 | 0.205 | 3.64% | 0.9987 | 1.088 | 0.371 |
|  | PW91PW91 | 0.340 | 0.489 | 0.326 | 0.116 | 0.274 | 4.56% | 0.9987 | 1.110 | 0.431 |
|  | BP86 | 0.341 | 0.489 | 0.326 | 0.116 | 0.275 | 4.57% | 0.9987 | 1.109 | 0.425 |
|  | B3LYP | 0.366 | 0.546 | 0.343 | 0.134 | 0.311 | 4.52% | 0.9983 | 0.906 | 0.358 |
|  | B3PW91 | 0.414 | 0.570 | 0.392 | 0.171 | 0.357 | 5.07% | 0.9988 | 1.115 | 0.384 |
|  | HSEH1PBE | 0.436 | 0.620 | 0.411 | 0.190 | 0.376 | 5.32% | 0.9985 | 1.120 | 0.400 |
|  | WB97XD | 0.453 | 0.680 | 0.418 | 0.205 | 0.395 | 5.30% | 0.9976 | 1.116 | 0.364 |
|  | PBE1PBE | 0.453 | 0.619 | 0.427 | 0.205 | 0.396 | 5.42% | 0.9988 | 1.122 | 0.389 |
|  | CAM-B3LYP | 0.468 | 0.669 | 0.435 | 0.219 | 0.414 | 5.41% | 0.9983 | 1.118 | 0.357 |
|  | LSDA | 0.473 | 0.633 | 0.454 | 0.223 | 0.398 | 6.03% | 0.9988 | 1.142 | 0.499 |
|  | LC-WPBE | 0.592 | 0.769 | 0.553 | 0.350 | 0.533 | 6.69% | 0.9990 | 1.145 | 0.398 |
|  | M6-2X | 0.926 | 1.074 | 0.869 | 0.857 | 0.869 | 10.01% | 0.9990 | 1.181 | 0.308 |

Table S6. Descriptors computed for ^1^H deviations for combinations of geometry, basis set, and NMR functional (C5-C8).

| Combination | **Functional** | **RMSE** | **MAX** | **MAE** | **MSE** | **ME** | **MAPE** | **R^2^** | **slope** | **intercept** |
| --- | --- | --- | --- | --- | --- | --- | --- | --- | --- | --- |
| TMT | B97D | 0.198 | 0.373 | 0.171 | 0.039 | 0.127 | 2.81% | 0.9975 | 1.070 | 0.342 |
|  | TPSSTPSS | 0.200 | 0.380 | 0.170 | 0.040 | 0.126 | 2.82% | 0.9972 | 1.085 | 0.319 |
|  | BP86 | 0.267 | 0.446 | 0.248 | 0.071 | 0.198 | 3.71% | 0.9979 | 1.104 | 0.330 |
|  | PW91PW91 | 0.274 | 0.456 | 0.254 | 0.075 | 0.203 | 3.81% | 0.9978 | 1.070 | 0.338 |
|  | B3LYP | 0.303 | 0.520 | 0.275 | 0.092 | 0.247 | 3.74% | 0.9973 | 1.180 | 0.386 |
|  | B3PW91 | 0.344 | 0.538 | 0.320 | 0.118 | 0.286 | 4.31% | 0.9980 | 1.105 | 0.367 |
|  | PBE1PBE | 0.379 | 0.585 | 0.352 | 0.143 | 0.320 | 4.65% | 0.9978 | 1.093 | 0.403 |
|  | LSDA | 0.395 | 0.578 | 0.376 | 0.156 | 0.326 | 5.11% | 0.9983 | 1.091 | 0.394 |
|  | HSEH1PBE | 0.403 | 0.609 | 0.372 | 0.162 | 0.350 | 4.73% | 0.9980 | 1.104 | 0.336 |
|  | CAM-B3LYP | 0.410 | 0.641 | 0.376 | 0.168 | 0.356 | 4.76% | 0.9975 | 1.099 | 0.360 |
|  | WB97XD | 0.439 | 0.610 | 0.412 | 0.193 | 0.383 | 5.20% | 0.9986 | 1.121 | 0.449 |
|  | LC-WPBE | 0.540 | 0.727 | 0.505 | 0.291 | 0.480 | 6.24% | 0.9986 | 1.116 | 0.372 |
|  | M6-2X | 0.848 | 1.144 | 0.782 | 0.719 | 0.782 | 9.19% | 0.9976 | 1.137 | 0.401 |
| PMT | TPSSTPSS | 0.267 | 0.430 | 0.252 | 0.071 | 0.201 | 3.74% | 0.9984 | 1.092 | 0.395 |
|  | B97D | 0.273 | 0.427 | 0.259 | 0.075 | 0.208 | 3.83% | 0.9985 | 1.094 | 0.401 |
|  | PW91PW91 | 0.350 | 0.528 | 0.335 | 0.123 | 0.278 | 4.76% | 0.9985 | 1.116 | 0.462 |
|  | BP86 | 0.350 | 0.527 | 0.335 | 0.123 | 0.278 | 4.76% | 0.9984 | 1.115 | 0.457 |
|  | B3LYP | 0.374 | 0.581 | 0.350 | 0.140 | 0.313 | 4.71% | 0.9980 | 1.108 | 0.392 |
|  | B3PW91 | 0.421 | 0.607 | 0.399 | 0.178 | 0.359 | 5.26% | 0.9985 | 1.121 | 0.420 |
|  | HSEH1PBE | 0.444 | 0.658 | 0.419 | 0.197 | 0.378 | 5.51% | 0.9981 | 1.126 | 0.435 |
|  | PBE1PBE | 0.460 | 0.655 | 0.435 | 0.212 | 0.397 | 5.62% | 0.9985 | 1.128 | 0.425 |
|  | WB97XD | 0.465 | 0.725 | 0.427 | 0.216 | 0.399 | 5.53% | 0.9967 | 1.122 | 0.404 |
|  | CAM-B3LYP | 0.477 | 0.704 | 0.444 | 0.227 | 0.416 | 5.63% | 0.9979 | 1.125 | 0.394 |
|  | LSDA | 0.483 | 0.675 | 0.464 | 0.233 | 0.403 | 6.24% | 0.9986 | 1.148 | 0.530 |
|  | LC-WPBE | 0.601 | 0.808 | 0.563 | 0.361 | 0.535 | 6.93% | 0.9987 | 1.152 | 0.438 |
|  | M6-2X | 0.924 | 1.065 | 0.864 | 0.853 | 0.864 | 9.87% | 0.9993 | 1.186 | 0.339 |
| TMP | B97D | 0.178 | 0.337 | 0.153 | 0.032 | 0.093 | 2.64% | 0.9973 | 1.068 | 0.362 |
|  | TPSSTPSS | 0.274 | 0.477 | 0.249 | 0.075 | 0.214 | 3.56% | 0.9974 | 1.083 | 0.338 |
|  | BP86 | 0.378 | 0.596 | 0.349 | 0.143 | 0.321 | 4.58% | 0.9976 | 1.102 | 0.351 |
|  | PW91PW91 | 0.176 | 0.331 | 0.152 | 0.031 | 0.096 | 2.64% | 0.9976 | 1.068 | 0.357 |
|  | B3LYP | 0.815 | 1.100 | 0.748 | 0.663 | 0.748 | 8.67% | 0.9978 | 1.179 | 0.406 |
|  | B3PW91 | 0.348 | 0.541 | 0.326 | 0.121 | 0.286 | 4.47% | 0.9979 | 1.103 | 0.388 |
|  | HSEH1PBE | 0.248 | 0.413 | 0.229 | 0.061 | 0.171 | 3.64% | 0.9979 | 1.091 | 0.422 |
|  | PBE1PBE | 0.241 | 0.403 | 0.222 | 0.058 | 0.165 | 3.54% | 0.9980 | 1.089 | 0.414 |
|  | LSDA | 0.343 | 0.532 | 0.322 | 0.118 | 0.282 | 4.41% | 0.9981 | 1.102 | 0.386 |
|  | CAM-B3LYP | 0.314 | 0.494 | 0.294 | 0.099 | 0.252 | 4.13% | 0.9981 | 1.097 | 0.380 |
|  | WB97XD | 0.366 | 0.534 | 0.350 | 0.134 | 0.292 | 4.93% | 0.9984 | 1.119 | 0.469 |
|  | LC-WPBE | 0.506 | 0.681 | 0.477 | 0.256 | 0.443 | 6.04% | 0.9987 | 1.135 | 0.422 |
|  | M6-2X | 0.407 | 0.566 | 0.386 | 0.166 | 0.349 | 5.02% | 0.9987 | 1.115 | 0.392 |
| PMP | TPSSTPSS | 0.254 | 0.403 | 0.240 | 0.064 | 0.186 | 3.63% | 0.9985 | 1.090 | 0.399 |
|  | B97D | 0.260 | 0.401 | 0.247 | 0.068 | 0.193 | 3.73% | 0.9987 | 1.092 | 0.405 |
|  | PW91PW91 | 0.336 | 0.502 | 0.323 | 0.113 | 0.263 | 4.66% | 0.9986 | 1.114 | 0.466 |
|  | BP86 | 0.336 | 0.500 | 0.322 | 0.113 | 0.264 | 4.66% | 0.9985 | 1.113 | 0.461 |
|  | B3LYP | 0.360 | 0.554 | 0.338 | 0.129 | 0.297 | 4.60% | 0.9981 | 1.107 | 0.395 |
|  | B3PW91 | 0.407 | 0.580 | 0.387 | 0.165 | 0.343 | 5.15% | 0.9987 | 1.119 | 0.423 |
|  | HSEH1PBE | 0.429 | 0.632 | 0.406 | 0.184 | 0.362 | 5.40% | 0.9982 | 1.124 | 0.438 |
|  | PBE1PBE | 0.445 | 0.629 | 0.422 | 0.198 | 0.381 | 5.51% | 0.9986 | 1.126 | 0.428 |
|  | WB97XD | 0.450 | 0.699 | 0.414 | 0.203 | 0.384 | 5.42% | 0.9968 | 1.120 | 0.405 |
|  | CAM-B3LYP | 0.461 | 0.677 | 0.431 | 0.213 | 0.400 | 5.51% | 0.9981 | 1.123 | 0.397 |
|  | LSDA | 0.468 | 0.648 | 0.451 | 0.219 | 0.388 | 6.13% | 0.9987 | 1.146 | 0.534 |
|  | LC-WPBE | 0.585 | 0.782 | 0.549 | 0.342 | 0.519 | 6.81% | 0.9987 | 1.150 | 0.442 |
|  | M6-2X | 0.910 | 1.052 | 0.850 | 0.827 | 0.850 | 9.69% | 0.9992 | 1.184 | 0.340 |

Table S7. Descriptors computed for ^13^C deviations for combinations C1-C4.

| Combination | **Functional** | **RMSE** | **MAX** | **MAE** | **MSE** | **ME** | **MAPE** | **R^2^** | **slope** | **intercept** |
| --- | --- | --- | --- | --- | --- | --- | --- | --- | --- | --- |
| TBT | B97D | 4.06 | 7.60 | 3.02 | 16.51 | 0.44 | 3.95% | 0.9867 | 0.984 | 2.381 |
|  | TPSSTPSS | 4.08 | 7.50 | 3.12 | 16.67 | 0.34 | 4.34% | 0.9865 | 0.979 | 2.874 |
|  | BP86 | 4.77 | 9.78 | 3.83 | 22.73 | 2.87 | 4.28% | 0.9885 | 1.007 | 2.004 |
|  | PW91PW91 | 5.14 | 10.28 | 4.21 | 26.44 | 3.47 | 4.44% | 0.9888 | 1.014 | 1.804 |
|  | B3PW91 | 5.95 | 12.20 | 4.98 | 35.35 | 4.41 | 4.46% | 0.9888 | 1.031 | 0.545 |
|  | PBE1PBE | 7.02 | 13.80 | 6.12 | 49.28 | 5.77 | 5.89% | 0.9891 | 1.036 | 1.391 |
|  | HSEH1PBE | 7.24 | 14.01 | 6.33 | 52.45 | 6.01 | 6.00% | 0.9891 | 1.039 | 1.266 |
|  | B3LYP | 7.38 | 14.53 | 6.43 | 54.54 | 6.07 | 6.25% | 0.9881 | 1.039 | 1.257 |
|  | LSDA | 8.29 | 14.20 | 7.29 | 68.73 | 7.21 | 5.94% | 0.9903 | 1.053 | 0.644 |
|  | WB97XD | 8.42 | 16.49 | 7.35 | 70.83 | 7.18 | 6.87% | 0.9881 | 1.050 | 1.024 |
|  | CAM-B3LYP | 9.44 | 18.09 | 8.25 | 89.09 | 8.25 | 7.25% | 0.9885 | 1.064 | 0.388 |
|  | LC-WPBE | 11.62 | 20.90 | 10.54 | 134.95 | 10.54 | 9.41% | 0.9883 | 1.076 | 1.170 |
|  | M6-2X | 20.90 | 26.18 | 19.93 | 436.81 | 19.93 | 16.78% | 0.9924 | 1.149 | 1.613 |
| PBT | B97D | 4.01 | 8.25 | 3.11 | 16.04 | -0.47 | 4.36% | 0.9873 | 0.972 | 3.006 |
|  | TPSSTPSS | 4.01 | 7.96 | 3.16 | 16.09 | -0.31 | 4.76% | 0.9873 | 0.969 | 3.551 |
|  | BP86 | 4.45 | 9.86 | 3.63 | 19.84 | 2.56 | 4.55% | 0.9892 | 0.998 | 2.754 |
|  | PW91PW91 | 4.68 | 10.29 | 3.87 | 21.89 | 2.98 | 4.46% | 0.9896 | 1.006 | 2.290 |
|  | PBE1PBE | 6.14 | 11.73 | 5.31 | 37.68 | 4.85 | 5.85% | 0.9896 | 1.024 | 1.910 |
|  | B3PW91 | 6.13 | 11.96 | 5.30 | 37.56 | 4.83 | 5.59% | 0.9894 | 1.020 | 2.353 |
|  | HSEH1PBE | 6.35 | 12.24 | 5.51 | 40.33 | 5.10 | 5.71% | 0.9896 | 1.027 | 1.826 |
|  | B3LYP | 7.01 | 13.38 | 6.12 | 49.10 | 5.75 | 6.38% | 0.9887 | 1.032 | 1.842 |
|  | WB97XD | 7.22 | 14.68 | 6.27 | 52.17 | 5.95 | 6.44% | 0.9885 | 1.034 | 1.731 |
|  | LSDA | 8.46 | 15.00 | 7.48 | 71.50 | 7.48 | 6.50% | 0.9908 | 1.050 | 1.336 |
|  | CAM-B3LYP | 9.49 | 17.78 | 8.35 | 90.04 | 8.35 | 7.76% | 0.9886 | 1.060 | 0.946 |
|  | LC-WPBE | 10.45 | 19.00 | 9.40 | 109.10 | 9.40 | 8.78% | 0.9888 | 1.064 | 1.491 |
|  | M6-2X | 19.38 | 24.34 | 18.41 | 375.64 | 18.41 | 15.90% | 0.9908 | 1.135 | 1.868 |
| TBP | B97D | 4.11 | 7.69 | 3.05 | 16.90 | 0.44 | 4.02% | 0.9863 | 0.984 | 2.438 |
|  | TPSSTPSS | 4.13 | 7.61 | 3.15 | 17.07 | 0.33 | 4.40% | 0.9862 | 0.979 | 2.928 |
|  | BP86 | 4.80 | 9.92 | 3.84 | 23.00 | 2.86 | 4.32% | 0.9882 | 1.007 | 2.057 |
|  | PW91PW91 | 5.17 | 10.42 | 4.22 | 26.70 | 3.46 | 4.48% | 0.9886 | 1.013 | 1.858 |
|  | B3PW91 | 5.87 | 12.19 | 4.87 | 34.42 | 4.26 | 4.32% | 0.9885 | 1.031 | 0.462 |
|  | PBE1PBE | 6.98 | 13.83 | 6.06 | 48.69 | 5.70 | 5.83% | 0.9889 | 1.035 | 1.358 |
|  | HSEH1PBE | 7.23 | 14.10 | 6.30 | 52.29 | 5.97 | 6.00% | 0.9888 | 1.038 | 1.288 |
|  | B3LYP | 7.37 | 14.62 | 6.40 | 54.39 | 6.02 | 6.25% | 0.9878 | 1.039 | 1.282 |
|  | WB97XD | 8.32 | 16.50 | 7.24 | 69.18 | 7.04 | 6.73% | 0.9878 | 1.053 | 0.674 |
|  | LSDA | 8.28 | 14.31 | 7.28 | 68.50 | 7.18 | 5.95% | 0.9901 | 1.050 | 0.954 |
|  | CAM-B3LYP | 9.87 | 18.94 | 8.65 | 97.46 | 8.65 | 7.63% | 0.9878 | 1.067 | 0.437 |
|  | LC-WPBE | 11.51 | 20.89 | 10.41 | 132.39 | 10.41 | 9.26% | 0.9881 | 1.076 | 1.111 |
|  | M6-2X | 20.98 | 26.40 | 20.01 | 439.96 | 20.01 | 16.95% | 0.9923 | 1.149 | 1.722 |
| PBP | B97D | 4.06 | 8.30 | 3.15 | 16.48 | -0.48 | 4.43% | 0.9870 | 1.020 | 2.406 |
|  | TPSSTPSS | 4.06 | 8.00 | 3.20 | 16.52 | -0.32 | 4.82% | 0.9869 | 0.968 | 3.612 |
|  | BP86 | 4.48 | 10.00 | 3.64 | 20.11 | 2.55 | 4.60% | 0.9889 | 1.031 | 1.892 |
|  | PW91PW91 | 4.70 | 10.42 | 3.87 | 22.11 | 2.96 | 4.50% | 0.9893 | 1.005 | 2.345 |
|  | PBE1PBE | 6.15 | 11.84 | 5.28 | 37.83 | 4.83 | 5.88% | 0.9891 | 1.049 | 1.388 |
|  | B3PW91 | 6.13 | 12.06 | 5.30 | 37.63 | 4.80 | 5.61% | 0.9892 | 0.998 | 2.818 |
|  | HSEH1PBE | 6.36 | 12.34 | 5.50 | 40.41 | 5.07 | 5.73% | 0.9893 | 1.034 | 1.750 |
|  | B3LYP | 7.01 | 13.48 | 6.11 | 49.15 | 5.72 | 6.40% | 0.9884 | 1.134 | 1.978 |
|  | WB97XD | 7.20 | 14.77 | 6.23 | 51.90 | 5.90 | 6.42% | 0.9881 | 1.060 | 0.971 |
|  | LSDA | 8.45 | 15.13 | 7.46 | 71.38 | 7.46 | 6.51% | 0.9906 | 1.064 | 1.503 |
|  | CAM-B3LYP | 9.46 | 17.86 | 8.30 | 89.50 | 8.30 | 7.73% | 0.9883 | 0.971 | 3.068 |
|  | LC-WPBE | 10.40 | 19.06 | 9.33 | 108.14 | 9.33 | 8.74% | 0.9885 | 1.026 | 1.873 |
|  | M6-2X | 19.40 | 24.52 | 18.43 | 376.53 | 18.43 | 15.98% | 0.9906 | 1.023 | 1.954 |

Table S8. Descriptors computed for ^13^C deviations for combinations C5-C8

| combination | **Functional** | **RMSE** | **MAX** | **MAE** | **MSE** | **ME** | **MAPE** | **R^2^** | **slope** | **intercept** |
| --- | --- | --- | --- | --- | --- | --- | --- | --- | --- | --- |
| TMT | B97D | 3.90 | 8.12 | 3.12 | 15.24 | -0.29 | 3.90% | 0.9877 | 0.979 | 2.316 |
|  | TPSSTPSS | 3.95 | 8.04 | 3.27 | 15.64 | -0.41 | 4.30% | 0.9875 | 1.070 | 0.342 |
|  | BP86 | 4.22 | 8.81 | 3.26 | 17.77 | 2.11 | 3.72% | 0.9893 | 1.001 | 1.945 |
|  | PW91PW91 | 4.53 | 9.30 | 3.64 | 20.52 | 2.71 | 3.88% | 0.9896 | 1.008 | 1.751 |
|  | B3PW91 | 5.18 | 10.60 | 4.29 | 26.80 | 3.59 | 3.81% | 0.9899 | 1.025 | 0.502 |
|  | PBE1PBE | 6.20 | 12.18 | 5.39 | 38.41 | 4.95 | 5.23% | 0.9902 | 1.029 | 1.366 |
|  | B3LYP | 6.53 | 12.85 | 5.68 | 42.61 | 5.22 | 5.58% | 0.9893 | 1.032 | 1.246 |
|  | WB97XD | 7.52 | 14.78 | 6.59 | 56.57 | 6.33 | 6.17% | 0.9894 | 1.043 | 1.029 |
|  | LSDA | 7.54 | 13.23 | 6.63 | 56.89 | 6.45 | 5.33% | 0.9908 | 1.048 | 0.592 |
|  | HSEH1PBE | 8.60 | 14.93 | 7.73 | 73.97 | 7.73 | 8.62% | 0.9902 | 1.032 | 3.786 |
|  | CAM-B3LYP | 9.00 | 17.12 | 7.88 | 80.99 | 7.88 | 6.96% | 0.9895 | 1.060 | 0.503 |
|  | LC-WPBE | 10.68 | 19.13 | 9.67 | 114.08 | 9.67 | 8.63% | 0.9896 | 1.069 | 1.198 |
|  | M6-2X | 19.82 | 24.21 | 18.90 | 393.00 | 18.90 | 15.77% | 0.9930 | 1.142 | 1.467 |
| PMT | BP86 | 4.06 | 8.88 | 3.29 | 16.45 | -1.22 | 4.38% | 0.9883 | 0.992 | 2.715 |
|  | TPSSTPSS | 4.02 | 8.56 | 3.33 | 16.12 | -1.06 | 4.75% | 0.9884 | 0.963 | 3.496 |
|  | B97D | 3.93 | 8.91 | 3.07 | 15.48 | 1.79 | 4.02% | 0.9900 | 0.966 | 2.970 |
|  | PW91PW91 | 4.10 | 9.33 | 3.30 | 16.78 | 2.21 | 3.94% | 0.9904 | 1.000 | 2.269 |
|  | B3PW91 | 4.97 | 9.60 | 4.25 | 24.68 | 3.51 | 4.59% | 0.9905 | 1.014 | 1.804 |
|  | PBE1PBE | 5.33 | 10.33 | 4.61 | 28.44 | 4.02 | 4.96% | 0.9907 | 1.017 | 1.897 |
|  | HSEH1PBE | 5.54 | 10.61 | 4.81 | 30.72 | 4.28 | 5.07% | 0.9907 | 1.020 | 1.817 |
|  | B3LYP | 6.15 | 11.68 | 5.39 | 37.81 | 4.90 | 5.73% | 0.9899 | 1.025 | 1.844 |
|  | WB97XD | 6.36 | 13.01 | 5.54 | 40.51 | 5.12 | 5.79% | 0.9897 | 1.028 | 1.746 |
|  | LSDA | 7.69 | 14.05 | 6.80 | 59.14 | 6.71 | 5.90% | 0.9913 | 1.044 | 1.315 |
|  | CAM-B3LYP | 8.55 | 16.00 | 7.53 | 73.05 | 7.48 | 7.03% | 0.9900 | 1.053 | 0.983 |
|  | LC-WPBE | 9.53 | 17.25 | 8.55 | 90.74 | 8.55 | 8.02% | 0.9902 | 1.057 | 1.511 |
|  | M6-2X | 18.34 | 22.43 | 17.43 | 336.42 | 17.43 | 15.01% | 0.9918 | 1.127 | 1.875 |
| TMP | B97D | 3.97 | 8.17 | 3.17 | 15.80 | -0.26 | 3.96% | 0.9872 | 0.979 | 2.331 |
|  | TPSSTPSS | 4.02 | 8.09 | 3.32 | 16.19 | -0.38 | 4.36% | 0.9870 | 0.974 | 2.803 |
|  | BP86 | 4.29 | 9.08 | 3.31 | 18.40 | 2.14 | 3.77% | 0.9889 | 1.002 | 1.957 |
|  | PW91PW91 | 4.60 | 9.57 | 3.69 | 21.18 | 2.74 | 3.94% | 0.9892 | 1.008 | 1.765 |
|  | B3PW91 | 5.25 | 10.80 | 4.33 | 27.53 | 3.62 | 3.86% | 0.9895 | 1.025 | 0.516 |
|  | PBE1PBE | 6.23 | 12.34 | 5.39 | 38.79 | 4.94 | 5.22% | 0.9898 | 1.029 | 1.342 |
|  | HSEH1PBE | 6.45 | 12.56 | 5.61 | 41.59 | 5.18 | 5.34% | 0.9898 | 1.032 | 1.226 |
|  | B3LYP | 6.57 | 13.03 | 5.69 | 43.14 | 5.22 | 5.58% | 0.9889 | 1.033 | 1.231 |
|  | WB97XD | 7.46 | 14.83 | 6.51 | 55.71 | 6.23 | 6.05% | 0.9891 | 1.043 | 0.926 |
|  | LSDA | 7.58 | 13.48 | 6.65 | 57.42 | 6.46 | 5.35% | 0.9905 | 1.048 | 0.583 |
|  | CAM-B3LYP | 8.97 | 17.22 | 7.83 | 80.45 | 7.81 | 6.88% | 0.9892 | 1.060 | 0.436 |
|  | LC-WPBE | 10.61 | 19.17 | 9.57 | 112.50 | 9.57 | 8.50% | 0.9894 | 1.069 | 1.105 |
|  | M6-2X | 19.95 | 24.51 | 19.02 | 397.83 | 19.02 | 15.94% | 0.9929 | 1.142 | 1.580 |
| PMP | BP86 | 4.13 | 8.96 | 3.33 | 17.06 | -1.20 | 4.44% | 0.9877 | 0.992 | 2.740 |
|  | TPSSTPSS | 4.09 | 8.63 | 3.37 | 16.72 | -1.04 | 4.81% | 0.9878 | 0.963 | 3.519 |
|  | B97D | 4.01 | 9.18 | 3.12 | 16.12 | 1.81 | 4.07% | 0.9895 | 0.966 | 2.992 |
|  | PW91PW91 | 4.17 | 9.59 | 3.34 | 17.39 | 2.22 | 3.99% | 0.9899 | 0.999 | 2.288 |
|  | B3PW91 | 5.03 | 9.78 | 4.29 | 25.34 | 3.52 | 4.63% | 0.9900 | 1.014 | 1.819 |
|  | PBE1PBE | 5.39 | 10.51 | 4.64 | 29.02 | 4.02 | 4.99% | 0.9902 | 1.017 | 1.905 |
|  | HSEH1PBE | 5.60 | 10.79 | 4.82 | 31.34 | 4.29 | 5.10% | 0.9903 | 1.020 | 1.827 |
|  | B3LYP | 6.20 | 11.88 | 5.41 | 38.49 | 4.91 | 5.75% | 0.9895 | 1.025 | 1.857 |
|  | WB97XD | 6.39 | 13.15 | 5.53 | 40.78 | 5.09 | 5.78% | 0.9893 | 1.027 | 1.729 |
|  | LSDA | 7.73 | 14.31 | 6.83 | 59.75 | 6.72 | 5.94% | 0.9909 | 1.044 | 1.333 |
|  | CAM-B3LYP | 8.56 | 16.15 | 7.53 | 73.31 | 7.47 | 7.03% | 0.9897 | 1.053 | 0.974 |
|  | LC-WPBE | 9.52 | 17.36 | 8.52 | 90.56 | 8.52 | 7.99% | 0.9899 | 1.057 | 1.490 |
|  | M6-2X | 18.41 | 22.68 | 17.49 | 338.94 | 17.49 | 15.11% | 0.9915 | 1.127 | 1.954 |

**Supporting Figures**


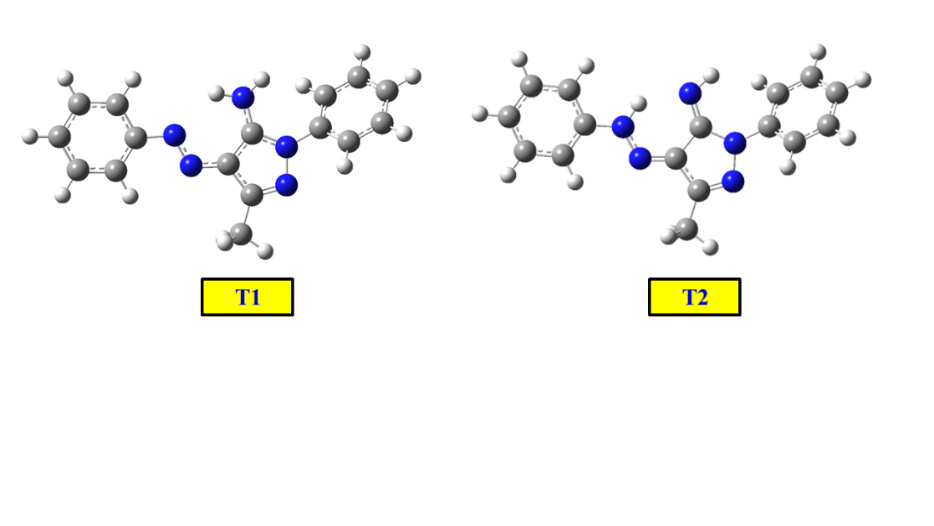


Figure S1. Optimized structures of the T1 and T2 tautomers of the 3-methyl-1-phenyl-4-(phenyldiazenyl)-1H-pyrazol-5-amine as obtained using B3LYP/6-311+G(2d,p) level of theory in acetonitrile using IEFPCM solvation model.


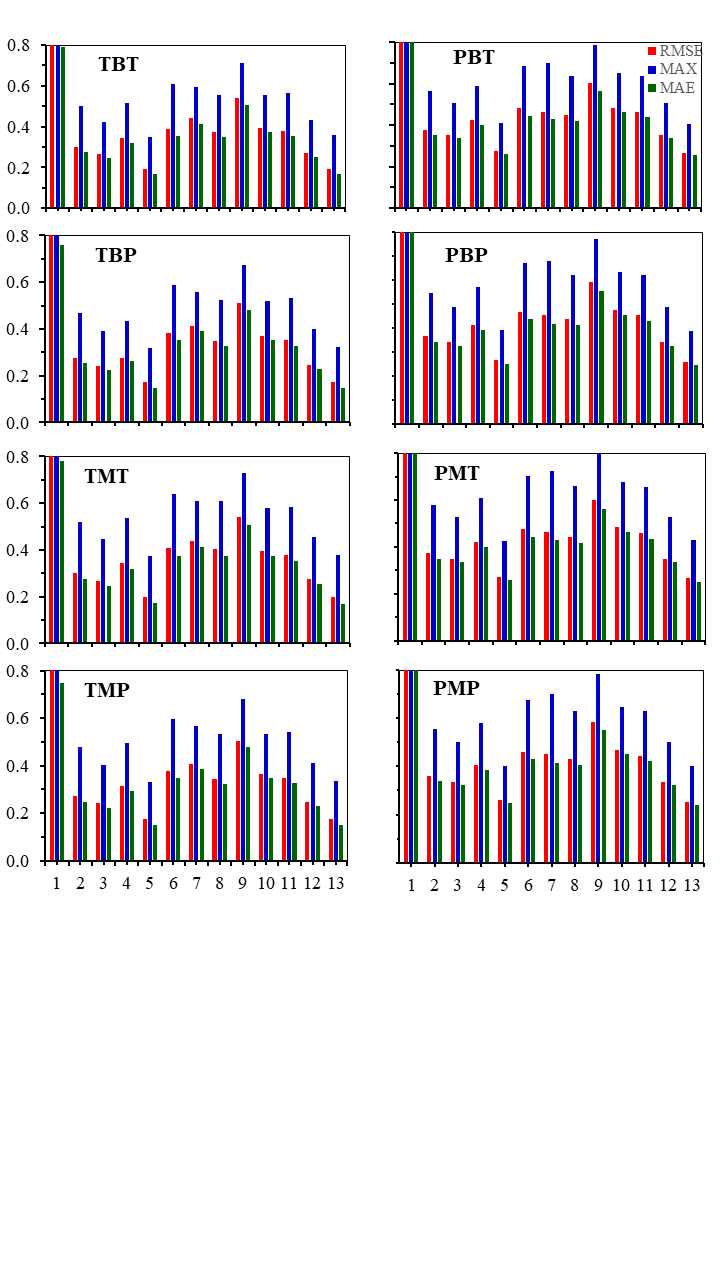


Figure S2. Graphical representation of the statistical descriptors (**RMSE** (red column) **MAX** (blue column), and **MAE** (dark red column) in ppm) computed for ^1^H-NMR chemical shifts deviations for all the tested combinations. Numbers stand for: 1: M06-2X, 2: B3LYP, 3: BP86, 4: B3PW1, 5: B97D, 6: CAM-B3LYP, 7: wB97XD, 8: HSEH1PBE, 9: LC-WPBE, 10: LSDA, 11: PBE1PBE, 12: PW91PW91, and 13: TPSSTPSS functionals (see Table S4).


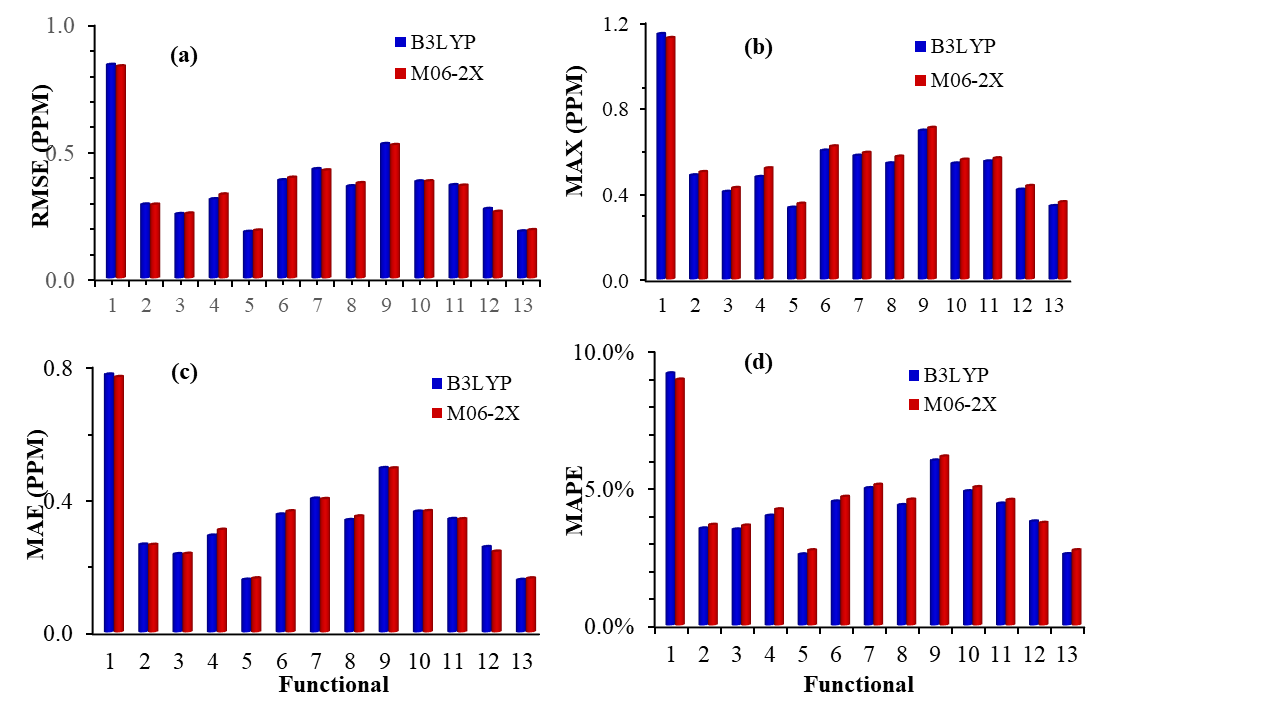


Figure S3. Graphical representation of the average descriptros (a) RMSE, (b) MAX, (c) MAE and (d) MAPE computed for the ^1^H-NMR chemical shifts of the 3-methyl-1-phenyl-4-(phenyldiazenyl)-1H-pyrazol-5-amine compound based on the B3LYP and M06-2X geometries.
